# Supplementary material for: Quantifying the financial burden of households’ out-of-pocket payments on medicines in India: a repeated cross-sectional analysis of National Sample Survey data, 1994–2014
Source: BMJ Open. 2018 May 31;8(5):e018020. doi: 10.1136/bmjopen-2017-018020 (PMC5988077; doi:10.1136/bmjopen-2017-018020)
Supplement: Supplementary data [file bmjopen-2017-018020supp001.pdf]

Annexure

Estimation of out-of-pocket expenditure indicators and related indicators

a. Out-of-pocket payments

Total out-of-pocket (OOP) payment has been defined as the summation of all kinds of direct expenditure on purchase of medical care including expenditure on family planning devices and transportation costs to access medical care by households either as inpatient or outpatient. According to the NSSO household questionnaire the main item of expenditure considered for inpatient and outpatient are presented in Table A-I.

Table A-I: Main items of expenditure considered for inpatient and outpatient in the NSSO questionnaire

**Inpatient**		**Outpatient**	
**Heads of expenditure**	**Item code in NSSO questionnaire**	**Heads of expenditure**	**Item code in NSSO questionnaire**
medicine	410	medicine	420
X-ray, ECG, pathological test, etc.	411	X-ray, ECG, pathological test, etc.	421
doctor's/surgeon's fee	412	doctor's/ surgeon's fee	422
hospital & nursing home charges	413	family planning devices	423
other medical expenses	414	other medical expenses	424
**Total inpatient**	**419=410-414**	**Total outpatient**	**429=420-424**

The reference period of inpatient and outpatient expenditure in the consumer expenditure surveys are 1 year and 1 month respectively. Based on the information presented in Table A-I, total inpatient expenditure, outpatient expenditure and total OOP expenditure were estimated by converting inpatient expenditure for one month. Accordingly, households with any OOP have been defined as households reporting positive OOP (OOP>0) either as inpatient or outpatient or both.

b. Per person monthly OOP and OOP share

Per person monthly OOP is defined as total monthly OOP divided by household size for each household.

The financial burden of health expenses by households has also been estimated in terms of OOP as a share of total household expenditure and alternatively as a share of total non-food expenditure of households..

$$Sh_{OOP} = T/\exp \quad \ldots\ldots\ldots\ldots\ldots\ldots\ldots\ldots \text{(A.1)}$$

Where, 'T' is total OOP payments and 'exp' is household total (non-food) expenditure by households.

c. Catastrophic payments and headcount

Further, OOP payments are defined as catastrophic when OOP payments as a portion of total household resources are in excess of a certain threshold. A household is said to have incurred catastrophic payments if T/exp>Z, where 'T' and OOP are the same as in equation (A.1) and 'Z' is a certain threshold. The latter is arbitrary and in general, estimates are presented for a range of values for z (5, 10, 15, 25 and 40 per cent).

Accordingly, the headcount ratio of catastrophic payment is calculated as follows:

$$cat_i = \frac{1}{n}\sum 1\left(\frac{T}{exp} > Z_i\right) \ldots\ldots\ldots\ldots\ldots\ldots\ldots\ldots\ldots\ldots\ldots\ldots\ldots \text{(A.2)}$$

Where, Cat_i is catastrophic headcounts of households with OOP share exceeding a threshold defined as 'i' per cent of total household total (non-food) expenditure, 1(.) is an indicator function, which takes the value 1 if T/exp>Zi is true and 0 otherwise; n is the number of households incurring expenditure on health for various thresholds; Z_1, Z_2, Z_3 …… are the respective thresholds of the OOP share.

d. Poverty headcount

The usual headcount ratio of poverty is calculated as:

$Gross\ HP = 1/n\ \Sigma\, 1\ (xi \leq PL)$ …………………………… (A.3)

where: 1(.) is a function, taking the value 1 if person belong to a household with consumption expenditure lower the value of poverty line value, x= Household total consumption expenditure; PL= Poverty Line and n is total population.

Headcount of poverty after deducting OOP from household consumption expenditure can be defined as:

Net HP = 1/n Σ 1 ((xi-T)≤ PL). ……………………………… (A.4)

where: T= per capita OOP

The OOP induced poverty headcount finally is estimated as: (*Gross HP - Net HP).*

e. Poverty gap

Poverty gap is defined as difference between values of poverty lines and household consumption expenditure for the poor as defined in the sub-section d above. Using the household level data poverty gap for the poor is estimated as follows:

Gross poverty gap = 1/n Σ 1 (xi- PL)| if i=poor …………………………… (A.5)

In equation A.5 'xi-PL' is the difference between household expenditure per person and poverty line. For all positive values of this difference mean gap is estimated. Finally, poverty gap net of OOP payments is estimated as follows:

Net poverty gap = 1/n Σ 1 ((xi-T)- PL)| if i=poor after netting OOP ……… (A.6)

Table A-II. Prevalence and average per episode total and medicine out of pocket payments by disease conditions in 2014

	Outpatient 15 days recall						Inpatient 365 days recall					
	Prevalence		Per episode		estimated monthly		Prevalence		Per episode		estimated monthly	
Ailment	Number of episodes	%	Total OOP	Drug	Total OOP	Drug	Number of episodes	%	Total OOP	Drug	Total OOP	Drug
Fever	2,71,43,431	22.71	488	280	975	561	74,12,043	12.95	8670	2329	713	191
TB/Filaria/Tetanus	11,08,425	0.93	524	287	1047	573	6,14,933	1.07	14731	4134	1,211	340
STD/HIV/AIDS	1,20,714	0.1	538	268	1076	536	88,935	0.16	6906	1633	568	134
Vector-borne	33,33,651	2.79	549	332	1097	663	22,65,189	3.96	10288	2460	846	202

Cancers	4,52,513	0.38	2527	1763	5054	3526	9,78,764	1.71	62297	14037	5,121	1,154
Blood disease	10,26,129	0.86	1322	731	2643	1463	8,10,752	1.42	15035	3650	1,236	300
Diabetes	1,17,55,081	9.84	683	456	1367	911	8,17,199	1.43	15746	4224	1,294	347
Other Metabolic	20,50,282	1.72	712	340	1423	679	2,60,707	0.46	15429	3600	1,268	296
Mental disorders	61,56,374	5.15	690	451	1380	902	24,87,836	4.35	26428	6685	2,172	549
Eye/Ear	24,67,286	2.06	950	454	1899	908	20,82,420	3.64	11350	1407	933	116
Cardiovascular diseases	1,55,65,223	13.02	645	449	1289	899	37,82,374	6.61	34167	6129	2,808	504
Respiratory diseases	1,69,58,670	14.19	478	328	955	656	21,40,762	3.74	14491	3325	1,191	273
Gastroenterlogy	77,13,330	6.45	809	434	1617	869	45,40,520	7.93	19587	4260	1,610	350
Skin	28,34,892	2.37	522	370	1043	740	3,80,346	0.66	12123	3514	996	289
Musculo-Skeletal	1,32,29,065	11.07	622	391	1244	782	19,66,211	3.44	24379	4677	2,004	384
Genito-Urinary	21,91,953	1.83	1183	747	2365	1494	28,01,133	4.89	27085	5094	2,226	419
Obstetric	3,64,060	0.3	1448	765	2896	1529	22,64,628	3.96	13050	2189	1,073	180
Injuries	19,93,646	1.67	1522	730	3045	1460	46,19,876	8.07	26242	6000	2,157	493
Others	28,92,298	2.42	655	426	1310	853	12,97,049	2.27	30196	6666	2,482	548
Childbirth	1,46,562	0.12	585	398	1169	797	1,56,17,000	27.29	8508	1729	699	142
