# Supplementary material for: Quantifying the financial burden of households’ out-of-pocket payments on medicines in India: a repeated cross-sectional analysis of National Sample Survey data, 1994–2014
Source: BMJ Open. 2018 May 31;8(5):e018020. doi: 10.1136/bmjopen-2017-018020 (PMC5988077; doi:10.1136/bmjopen-2017-018020)
Supplement: Author's manuscript [file bmjopen-2017-018020.draft_revisions.pdf]

BMJ Open is committed to open peer review. As part of this commitment we make the peer review history of every article we publish publicly available.

When an article is published we post the peer reviewers' comments and the authors' responses online. We also post the versions of the paper that were used during peer review. These are the versions that the peer review comments apply to.

The versions of the paper that follow are the versions that were submitted during the peer review process. They are not the versions of record or the final published versions. They should not be cited or distributed as the published version of this manuscript.

BMJ Open is an open access journal and the full, final, typeset and author-corrected version of record of the manuscript is available on our site with no access controls, subscription charges or pay-per-view fees (http://bmjopen.bmj.com).

If you have any questions on BMJ Open's open peer review process please email info.bmjopen@bmj.com

BMJ Open

Financial burden of medicine out of pocket payments: Implications for living status of Indian households

Journal:	*BMJ Open*
Manuscript ID	bmjopen-2017-018020
Article Type:	Research
Date Submitted by the Author:	03-Jun-2017
Complete List of Authors:	Selvaraj, Sakthivel; Public Health Foundation of India,<br>Farooqui, Habib; Public Health Foundation of India,<br>Karan, Anup; Indian Institute of Public Health, Delhi (IIPHD), Public Health Foundation of India,
**Primary Subject Heading**:	Health economics
Secondary Subject Heading:	Health policy, Health economics
Keywords:	out of pocket, poverty, catastrophe, medicine

SCHOLARONE™ Manuscripts

For peer review only

Title page

Title of the article:

Financial burden of medicine out of pocket payments: Implications for living status of Indian households

Full name, postal address, e-mail and telephone number of the corresponding author:

Dr. Habib Hasan Farooqui, MD
Associate Professor
Address: Public Health Foundation of India, Plot No. 47, Sector 44, Institutional Area, Gurugram, India -122001
Email: drhabibhasan@gmail.com
Phone: +91 9999984673

Full name, department, institution, city and country of all co-authors:

Dr. Sakthivel Selvaraj, PhD
Director,
Address: Public Health Foundation of India, Plot No. 47, Sector 44, Institutional Area, Gurugram, India -122001

Dr. Habib Hasan Farooqui, MD
Associate Professor
Address: Public Health Foundation of India, Plot No. 47, Sector 44, Institutional Area, Gurugram, India -122001

Dr. Anup Karan, DPhil
Associate Professor,
Address: Public Health Foundation of India, Plot No. 47, Sector 44, Institutional Area, Gurugram, India -122001

Word count, excluding title page, abstract, references, figures and tables;
3582 words,

For peer review only

Financial burden of medicine out of pocket payments: Implications for living status of Indian households

Objective: The objective of this research is to generate new evidence on financial implications of medicine out of pocket (OOP) payments for households. We also report disease conditions separately in outpatient and inpatient care, which caused significant increase in OOP expenses.

Setting: All Indian States including Union Territories, 1993-94 to 2014

Design: Repeated cross section analysis

Data: Secondary data of nationwide Consumer Expenditure Surveys for the years 1993-94, 2004-05 and 2011-12 and one wave of Social Consumption: Health for the year 2014 from National Sample Survey Organisation.

Outcome measure : Total out-of-pocket expenditure on healthcare and medicine out-of-pocket expenditure on healthcare.

Results: Total and medicine out-of-pocket were estimated to be 6.77% [CI: 6.70%, 6.84%] and 4.49% [4.45%, 4.54%] of total consumption expenditure in the year 2011-12 which marked significant increase since 1993-94. These proportions were 11.46% [CI: 11.36%, 11.56%] and 7.60% [CI: 7.54%, 7.67%] respectively of non-food expenditure in the same year. Total and medicine OOP were catastrophic for 17.9% [CI: 17.7%, 18.2%] and 11.2% [11.0%, 11.4%] households respectively in 2011-12 at the 10% of total consumption expenditure threshold implying 29 million households incurred catastrophic out-of-pocket payments in the year 2011-12. Further medicine out-of-pocket pushed 3.09% [CI: 2.99%, 3.20%], implying 38 million, persons into poverty in the year 2011-12. Among the main diseases which caused major out-of-pocket payments are cancers, cardiac, geneto-urinary and metal disorder conditions. Average monthly medicine OOP was invariably higher in outpatient care compared to inpatient care.

Conclusions: Government intervention in terms of public provisioning of free medicine has potential to significantly reduce the medicine related OOP as well as total OOP.

Key words: India, out of payment, medicine, catastrophe, poverty

Article summary

Strengths and limitations of this study

1. The study used multiple points of time and nationally representative data set to highlight the burden of medicine out-ofpocket expenditure incurred by households;
2. This study is the first one to produces evidence on catastrophic and poverty impact of out-of-pocket payments for medicine
3. The paper links medicine out-of-pocket payments by households with disease conditions and identify types of disease conditions which trigger medicine out-of-pocket payments
4. The study also highlights for the first time that out-of-pocket payments on medicines in outpatient care is far greater burden for households than expenditure on hospitalisation care
5. The study has limitations in terms of using arbitrary cut-off for measuring catastrophic payments
6. Self-reported disease conditions and related out-of-pocket expenditure is an obvious limitation of the study.

For peer review only

Background

Households' in India bear significant financial burden due to treatment, as prepayment and risk pooling mechanisms are inadequate. Since both government funding and social health insurance contributions are insufficient to meet health care needs of households, over three-fourth of overall health care payments are paid out-of-pocket (OOP) at the point of service delivery and medicine purchase account for the single largest component of these payments[1]. Such spending has dominated over the years, which has pushed significant proportion of population impoverished while a higher proportion of households have incurred catastrophic spending due to OOP payments[2-4].

The unpredictable and catastrophic nature of illness can throw households into the vortex of poverty. The predicament of high OOP could result in liquidation of assets, heavy borrowing, running down savings, etc. Evidence suggests that households' OOP expenditure pulls down a vast chunk of population below poverty line[2 5-8]. Similarly, recent findings documented the burden of catastrophic spending of health care on households owing to high OOP share to total households' resources [6 9 10]. In the Indian context, several studies in the past confirmed high OOP in general and OOP on medicine in particular. Estimates of impoverishment due to OOP vary widely depending upon the methods and year in which the estimates were made. The number of households falling below poverty line was in the range of 32-39 million per annum during the period 2004 [2 5-7]. Such evidence is no surprise given the fact that in India, OOP expenditures are approximately three quarters of total health expenditure [1 11].

Available evidence reveals that large proportions of population belonging to poor households forego formal treatments owing to family budget constraint. Further, it confirms earlier findings that a substantial burden of OOP is due to outpatient care while the financial burden caused by inpatient care episodes are to the extent of little over one-fifth, and the remaining four-fifth due to outpatient care visits [5]. Another study demonstrated that medicines purchase alone constituted over 70 percent of overall OOP payments. Further, it showed that by simply removing OOP payments for inpatient care resulted in a negligible decline in poverty headcount ratio and poverty gap. Interestingly, the research also demonstrated that by deducting OOPs payments for outpatient care or medicines, the percent of people falling below poverty worked out to just 0.5% [12].

Using the method of impoverishment to measure affordability, one study assessed the impoverishment effect of medicines purchases by households in 16 low-and-middle income economies [13]. Comparing four key medicine prices to household income in 16 nations, and using World Bank poverty levels of US$ 1.25 or US$ 2 per day, the study concluded that a substantial number of people are brought to bear financial burden due to unaffordability of medicines. In Philippines, it was pointed out that an originator brand atenolol purchase by individuals would push an additional 22% of population below the US$ 1.25 per day

For peer review only

measurement while even a generic equivalent of atenolol was likely to push about 7% of population below poverty levels [14].

Available evidence, both global and Indian, provides insights about the incidence of catastrophic payments and impoverishment impact of a high and rising households OOP. The literature on equity dimensions involving both catastrophe and impoverishment has attempted to address complex methodological and statistical approaches in measurements. The focus of this research, on the other hand, is to explore the consequences of high households' medicines spending, since a large share of households' OOP is often incurred on purchasing drugs. Further, we attempt to investigate which disease conditions are contributing to high financial burden on households' medicines spending. We attempted to answer - what is the contribution of medicine OOP by households in total OOP payments, catastrophic and poverty headcounts? And which disease conditions cause relatively higher financial disruption in the living status of households?

Materials and methods

Data

The study used secondary data from three waves of nationally representative 'Consumer Expenditure Surveys' (CES): 1993-94, 2004-05 and 2011-12, conducted by the National Sample Survey Organisation (NSSO). In addition, health and morbidity survey (HMS) 2014 of the NSSO was used for disease level classifications of OOP burden. While the sample of size of CES varied between 100,000 to 125,000 thousand households across different rounds, the sample size in HMS 2014 was approximately 72,000 households.

The CES collect socioeconomic and demographic information on households, but its key focus is on household spending on roughly 350 food and non-food items. Out-of-pocket medical expenses incurred by households are separately recorded for inpatient and outpatient services. The recall periods are one-year and 30-days for inpatient and outpatient expenses, respectively. HMS collects detailed information on morbidity types, utilisation pattern and expenditure by households incurred due to utilisation of healthcare. HMS too separately records expenditure for inpatient and outpatient. However, unlike in CES the recall period for outpatient in HMS is 15 days.

Both CES and are repeated cross-section surveys and are representative at the national and state levels. In most cases, all districts of a state are included for sampling purposes. Households in CES are sampled evenly in quarterly sub-rounds beginning on 1 July and ending on 30 June of the following year, with equal numbers of households allotted in each quarterly sub-round, to address seasonality. In HMS, survey was completed in only two sub-rounds starting January1 to June 30, 2014. All estimates in the present paper are sample weighted.

For peer review only

Outcome indicators

Using CES we estimated 4 household-level indicators of the financial burden of illness: i) per household member monthly OOP spending on medicines (inflation-adjusted), ii) OOP spending on medicines as a share of total household and non-food spending, and iii) percentage of households reporting catastrophic payments on medicines and iv) percentage of households slipping onto poverty after netting out medicines OOP from households' total consumption expenditure. Instead of sticking to a particular threshold, we considered a range of thresholds for assessing catastrophic expenditure [2 4 9 15]. We also considered alternative thresholds as medicine OOP spending as a share of household non-food expenditure. For OOP induced poverty estimates we used two different poverty lines: i) Indian official state-specific poverty line[16] and ii) international poverty line based on US $ 1 per day per person adjusted to US $ 1.9 purchasing power parity (PPP) per day per person for the year 2011-12[17]. Details of the method used for catastrophic and poverty estimates are presented in Appendix I.

In addition, we used NSSO 2014 HMS data for estimating disease level total and medicine OOP spending separately for inpatient and outpatient. Unlike CES, OOP spending in NSSO 2014 has not been recorded as part of the total household consumption expenditure and instead of estimating disease-wise catastrophic headcount, we present distribution of disease conditions based on incidence of occurrence and range of OOP spending separately for outpatient (15 days recall converted for 30 days) and inpatient (365 days recall converted for 30 days). This helped identifying disease conditions, separately for outpatient and inpatient, which are high frequency of occurrence and greater incidence of OOP spending.

Results

First, we present basic financial burden indicators for the years 1993-94, 2004-05 and 2011-12 in Table 1. Over 80 percent of populations are reportedly spending out-of-pocket while seeking treatment, during 2011-12. The proportion of population reporting any OOP payments have increased sharply from about 60 percent during 1993-94 to 80 percent in 2011-12. In respect to medicines spending, approximately every OOP spending is associated with expenditure on medicines. There was a significant increase (more than 50%) in household's total consumption expenditure in real terms from INR 517 in 1993-84 to INR 794 in 2011-12. However, during the same period total OOP increased by more than 100% from INR 26 in 1993-94 to INR 54 in 2011-12 in real terms. The increase in OOP on medicine has been more than 70% during the same period. Consequently, the share of spending on health from households' overall consumption expenditure have registered sharp increase during the past two decades, from a moderate 4.8 percent during 1993-94 to nearly seven percent in 2011-12. If we were to net out food expenditure from overall household spending, which are considered a necessity, the share of health spending remained stagnant

For peer review only - http://bmjopen.bmj.com/site/about/guidelines.xhtml

but as high as 11-12 percent during the period under consideration. It may be observed that in 2011-12 medicines alone contributed up to 67% of the total OOP.

Table 1: **Financial burden indicators, India, 1993-94, 2004-05 and 2011-12**

**Financial burden indicators**	**1993-94**	**2004-05**	**2011-12**
**Percentage households reporting oops**			
total oops	59.2 [58.9, 59.5]	64.4 [64.2, 64.7]	80.5 [80.2, 80.7]
medicines oops	57.5 [57.3, 57.8]	63.6 [63.3, 63.8]	79.0 [78.8, 79.3]
**Monthly per capita expenditure (INR)**			
total consumption expenditure (current prices)	317 [315, 318]	712 [708, 715]	1627 [1617, 1636]
total consumption expenditure (constant 1999-2000 prices)	517 [ ]	619 [616, 622]	794 [790, 799]
total out-of-pocket expenditure (current prices)	15.7 [15.4, 16.0]	41.8 [41.1, 42.6]	111.2 [109.1, 113.3]
total out-of-pocket expenditure (constant 1999-2000 prices)	25.59 [ ]	36.3 [35.7, 37.0]	54.3 [53.3, 55.3]
medicines out-of-pocket expenditure (current prices)	12.8 [12.6, 13.0]	29.8 [29.3, 30.2]	73.9 [72.5, 75.3]
medicines out-of-pocket expenditure (constant 1999-2000 prices)	20.86 [ ]	26.0 [25.6, 26.4]	36.1 [35.5, 36.8]
**Share of health to total household expenditure (%)**			
share of total out-of-pocket expenditure	4.84 [4.78, 4.91]	5.78 [5.72, 5.83]	6.77 [6.70, 6.84]
share of total medicines out-of-pocket expenditure	3.93 [3.87, 3.98]	4.10 [4.06, 4.14]	4.49 [4.45, 4.54]
**Share of health to non-food household expenditure (%)**			
share of oops to non-food expenditure	12.37 [12.20, 12.55]	10.82 [10.72, 10.91]	11.46 [11.36, 11.56]
share of medicines oops to non-food expenditure	10.02 [9.88, 10.17]	7.68 [7.62, 7.75]	7.60 [7.54, 7.67]

Note: numbers in brackets are 95% confidence interval

The households' financial burden on account of distress in OOP payments leads to i) impoverishment and ii) catastrophe. In Table 2 we present a set of catastrophic thresholds measured as a share of OOP to total household consumption expenditure and non-food expenditure. Estimates are presented for both overall OOP as well as OOPs underlying medicines expenditure of households. Further, we define catastrophic measures at different thresholds.

For peer review only - http://bmjopen.bmj.com/site/about/guidelines.xhtml

Table 2: Percentage of households incurring catastrophic payments with respect to total OOP and medicines spending, India, 1993-94, 2004-05 and 2011-12

**Financial Health Equity Measurements**	1993-94	2004-05	2011-12	Estimated number of households (2011-12)
**Thresholds using on total household expenditure**				
total OOP > 5%	26.9 [26.6, 27.1]	28.7 [28.5, 30.0]	35.3 [35.0, 35.6]	9,01,07,225
total OOP > 10%	13.9 [13.8, 14.2]	14.6 [14.4, 14.8]	17.9 [17.7, 18.2]	4,56,91,766
total OOP > 25%	3.9 [3.8, 4.0]	3.5 [3.4, 3.6]	4.3 [4.2, 4.4]	1,09,76,234
medicines OOP > 5%	23.3 [23.0, 23.5]	23.4 [23.2, 23.6]	27.0 [26.7, 27.2]	6,89,20,540
medicines OOP > 10%	11.5 [11.3, 11.7]	10.2 [10.2, 10.4]	11.2 [11.0, 11.4]	2,85,89,261
medicines OOP > 25%	02.9 [2.8, 2.9]	1.6 [1.5, 1.7]	1.8 [1.7, 1.9]	45,94,703
**Thresholds using on non-food expenditure**				
total OOP > 5%	47.8 [47.5, 48.1]	46.5 46.2, 46.8]	53.5 [53.2, 53.8]	13,65,64,775
total OOP > 10%	34.8 [34.6, 35.1]	31.0 [30.7, 31.2]	34.9 [34.7, 35.2]	8,90,86,180
total OOP > 25%	16.7 [16.5, 16.9]	11.4 [11.2, 11.5]	11.9 [11.7, 12.1]	3,03,76,090
total OOP > 40%	9.7 [9.5, 9.9]	4.7 [4.6, 4.9]	4.9 [4.8, 5.0]	1,25,07,802
medicines OOP > 5%	44.7 [44.4, 45.0]	42.5 [42.2, 42.8]	46.4 [46.1, 46.7]	11,84,41,225
medicines OOP > 10%	31.2 [31.0, 31.5]	25.5 [25.3, 25.7]	26.1 25.9, 26.4]	6,66,23,189
medicines OOP > 25%	13.9 [13.7, 14.1]	7.1 [7.0, 7.3]	6.3 [6.1, 6.4]	1,60,81,459
medicines OOP > 40%	7.8 [7.6, 7.9]	2.2 [2.1, 2.3]	1.8 [1.7, 1.9]	45,94,703

For peer review only

At the lowest cut-off level of five percent, over one-third of Indian households incurred OOP payments in 2011-12. This percentage was lower in 1993-94 (27%) and 2004-05 (28%). At the 25% threshold of total household expenditure, over 4% households reported incurring OOP in 2011-12, which again registered increasing trend during the last two decades. This essentially implies approximately 11 million Indian households in 2011-12 incurred OOP payments, which is more than 25% of their total household expenditure. Out of these more than 4.4 million households incurred such payments only on account of purchase of medicines. At a lower threshold of 10% of THE, the number of households facing catastrophe is approximately 46 millions, of which 29 million households incurred catastrophe on account of OOP on medicines. Considering only non-food expenditure of households as the basic living status variable, approximately similar number of households incurred medicine OOP in 2011-12, which is as high as 40% of their non-food expenditure.

Table 3: Impoverishment Indicators due to Households' OOP on Medicines, India, 1993-94, 2004-05 and 2011-12

	1993-94	2004-05	2011-12	Estimated population (2011-12)
**Using national poverty line***				
Gross Headcount	45.32 [45.03, 45.61]	37.85 [37.58, 38.12]	22.17 [21.92, 22.43]	27,19,03,356
Headcount net of total OOP	49.52 [49.22, 49.81]	42.68 [42.40, 42.95]	26.65 [26.38, 26.92]	32,68,48,193
Total OOP induced poverty	4.20 [4.07, 4.30]	4.83 [4.71, 4.94]	4.48 [4.35, 4.60]	5,49,44,837
Headcount net of medicine OOP	48.91 [48.61, 49.20]	41.54 [41.27, 41.82]	25.27 [25.00, 25.53]	30,99,23,221
Medicine OOP induced poverty	3.59 [3.47, 3.69]	3.69 [3.59, 3.80]	3.09 [2.99, 3.20]	3,78,97,220
**Using international poverty line****				
Gross Headcount	40.96 [40.67, 41.24]	33.07 [32.81, 33.34]	18.37 [18.13, 18.61]	22,52,98,360
Headcount net of total OOP	44.92 [44.63, 45.21]	37.38 [37.11, 37.65]	22.41 [22.16, 22.67]	27,48,46,829

Total OOP induced poverty	3.96 [3.85, 4.08]	4.31 [4.19, 4.42]	4.04 [3.92, 4.16]	4,95,48,469
Headcount net of medicine OOP	44.35 [44.06, 44.64]	36.34 [36.08, 36.61]	21.37 [21.11, 21.62]	26,20,91,778
Medicine OOP induced poverty	3.39 [3.29, 3.50]	3.27 [3.17, 3.68]	2.99 [2.89, 3.10]	3,66,70,773

Notes: based on Tendulkar Committee methods; ** using USD 1.90 PPP at 2011-12 prices and mixed recall period of household consumption expenditure

Next, we present implications of OOP payments on poverty estimates. To facilitate interpretation, we present 3 basic indicators: i) gross headcount – percentage of population below poverty line, ii) headcount net of OOP – percentage of population below poverty line after netting out OOP from household consumption expenditure and iii) OOP induced poverty which is the difference of the first two reflecting rise in poverty ratio owing to OOP. The last two indicators are presented separately for total OOP and medicine OOP. All these indicators are estimate using Indian official poverty line (Tendulkar Committee method) and international poverty line of US $ 1.90 PPP.

The difference in mean headcount measure of gross and net poverty ratio, reflects the percentage of population falling below poverty line because of households' OOP on health care. The percentage of headcount of households impoverished due to OOP was 3.97 percent during 1993-94, which increased to 4.30 percent in 2004-05 while in 2011-12 it was at 4.04 percent, as per international poverty line. In terms of Indian state-specific official poverty line, it may be observed that owing to OOP the percentage of households falling below poverty line increased from 4.19 percent in 1993-94 to 4.48 percent in 2011-12. This implies that close to 55 million persons in 2011-12 were pushed into poverty mainly because of OOP payments to healthcare. Out of this approximately 38 million became poor only because OOP payments on purchasing medicines. Using the same measurement, the headcount measure for households OOP on medicines slightly declined from 3.58 percent in 1993-94 to 3.09 percent during 2011-12 using the international poverty line. Two trends stand out clearly from the findings. Impoverishment arising out of households' OOP is rather high and continued to be so during the last two decades. Moreover, the burden of impoverishment arising out of households' OOP on medicines appears to be highest, where three fourth of all health impoverishment is due to medicines spending by households.

OOP expenditure by disease conditions

We also conducted a disaggregated analysis on disease wise expenditure not only with reference to total OOP and medicine OOP but also by type of care - inpatient versus out patient. Most common health condition for seeking outpatient care and inpatient care was

For peer review only

fever (22.7%) and childbirth (22.3%). In addition, our estimates suggest that cancer had the highest monthly total OOP per capita both for inpatient (INR 5054) and outpatient (INR 5121) care followed by injuries (INR 3045) for outpatient care and cardiac events (INR 2808) for inpatient care.

Since, medicines OOP contribute significantly to catastrophe of households as highlighted above, we mapped disease wise expenditure, frequency of utilization and type of care to generate a holistic scenario to explain how not only hospitalization but outpatient care for majority of the disease also leads to catastrophe and impoverishment (Figure 1). For example, our estimates suggest that per capita medicine OOP for cancer care was significantly higher in outpatient care as compared to the inpatient care. However, as far as total OOP is concerned, it is almost similar and high both for inpatient and outpatient. In contrast, in case of cardio, medicine OOP is similar both for inpatient and outpatient but total OOP is significantly higher for inpatient treatment compared to outpatient treatment. In case of gastro, however, both medicine OOP and total OOP both are higher for outpatient compared to inpatient treatment. Similarly, for mental disorder medicine OOP is higher for outpatient compared to inpatient but total OOP is almost similar both for outpatient and inpatient. In general, average monthly medicine OOP was consistently higher for outpatient care as compared to inpatient cares for majority of disease condition and coupled with higher frequency for outpatient care this is resulting into high incidence of catastrophe. The catastrophic nature of outpatient expenditure is also reflected at the household level.

We plotted outpatient and inpatient total OOP and medicine OOP in relation to households 'usual' consumption expenditure (Figure 2). For a number of households, average monthly outpatient expenditure is not only significantly higher in relation to household's non-medical consumption expenditure but also frequency of such events is higher in outpatient care as compared to inpatient care. The concentration of red (total OOP) and green (medicine OOP) spikes above the consumption expenditure on the right hand side of the graph reflects this scenario succinctly.

Discussion and conclusion

We demonstrated through a well-defined approach of measuring health cost burden on households that medicines OOP payments by households leads to impoverishment and catastrophe. Although evidence exist both in global and Indian literature on catastrophic and impoverishing nature of household's OOP, to the best of our knowledge this research work is the first attempt to capture impoverishing effect of OOP payments on account of medicine OOP in both outpatient and inpatient setting involving disease conditions. We observed that households' OOP is not only high in India but continues to remain so for over two decades. Also, the spending on medicines accounted for the largest share of OOP, accounting for over two-thirds of households' payments on health care. Applying a 10%

For peer review only

threshold of OOPs on overall consumption expenditure, an estimated 18 percent of Indian households appear to suffer financial catastrophe. In respect to medicines OOP, at similar threshold, an estimated 11 percent of Indian populations are reported to be incurring catastrophe. This essentially implies that an estimated 46 million households faced catastrophic expenditure on healthcare in the year 2011-12, if we consider 10% threshold of household consumption expenditure as catastrophe. Out of this 46 million, 29 million households incurred such expenditure mainly on account of purchasing medicines. Impoverishment of households arising out of households' OOP is high and continues to be so during the last two decades. Measured both by international poverty criteria as well as Indian state-specific official poverty lines, the burden of impoverishment due to households' OOP on medicines appears to be significantly high, where three fourth of all health impoverishment is due to medicines spending. An estimated 55 million population were pushed into poverty because they had to pay for healthcare. Medicines purchasing alone contributed to approximately 38 million of this increase in impoverishment.

Literature suggest that in India, on an average, the government spending on medicines to be around 10 percent, several state governments end up spending even less than five percent of public spending on health care [18].. In addition, except for a couple of Indian states, drug procurement and distribution system is ineffective leading to acute shortages of key essential medicines and chronic stock-outs in public health facilities[19-22]. This situation has resulted in physical unavailability of medicines. Drawing evidence from large sample surveys for the period from 1986-87 to 2004, it is reported the physical barrier to access to key essential medicines have worsened during this period. Supply of free drugs in government health system in the outpatient care setting, declined sharply from about 18 percent in 1986-87 to five percent in 2004. For the same period, drugs prescribed during hospitalization for free also declined significantly from one-third to about nine percent[18].. As a result, it is pointed out "the number of hospitalization episodes in which an ailing population paid out-of-pocket (OOP), has risen dramatically from about 41 percent to close to 72 percent".

Further, it was observed that from the period spanning mid-1990s to 2004, patients visiting government health facilities did not receive medicines in over one-fourth of outpatient episodes[23]. In view of inadequate availability of medicines in government health facilities, households' end up accessing private facilities. Evidence suggests that trend have sharpened in the last couple of decades. For instance, the percentage of population accessing private facilities for inpatient and outpatient treatment has increased significantly between 1986-87 and 2004, households accessing private hospital care increased from around 40 percent to nearly 60 percent in rural India while urban India reported increase from 40 percent to 68 percent[24]. For similar period, outpatient care visits in private facilities remained high at around 75 percent in 1986-87 in rural India and 73 percent in urban India stepped up to 78 percent and 80 percent respectively for rural and urban India [25].

For peer review only

Although public sector gained some ground by increasing its share in total outpatient care, private sector continue to dominate both in outpatient and inpatient care in India[23]. As an increasing share of households access private health facilities, private retail pharmacies have become a major source of supply of key essential medicines. While availability of medicines may per se not a challenge, affordability appears to act as barrier[26] . Thus, pricing of medicines and regulation around retail medicine prices becomes critical in improving affordability and consequently reducing medicine induced OOP burden. Although India had a progressive retail price cap policies since 1979, but over the years a policy of deregulation was followed[27]. In 2013, the Government of India promulgated the Drugs Price Control Order, 2013 (DPCO, 2013) which primarily brought all essential drugs, based on National List of Essential Medicines, 2011, under price cap[28]. However, the policy has moved from cost based pricing to market-based pricing mechanism. An evaluation of new price regulation has highlighted that, while few of the molecules analyzed (37) had an increase in sales volume attributable to DPCO, majority of the molecules (52) had a negative impact on their sales volume due to DPCO. Overall, the DPCO may have had a negative impact in terms of sales volume of medicines under price control[29]. Given that the sales volume of price controlled medicines has gone down, households OOP spending may increase on account of high medicine price in unregulated market.

Further, to improve access to health care and to provide financial risk protection to households, the central government and several state governments have been implementing a publicly-funded health insurance programs since 2007, whose primary aim was to provide cashless treatment to economically vulnerable households for hospitalization episodes. Emerging evidence from micro as well as macro level studies point to a trend where such insurance schemes appear to have improved access to hospital care but have been ineffective in preventing catastrophe and impoverishment to households[4 30 31]. These evidence are in line of our findings that hospitalization based treatment constitute only small part of the India's morbidity burden and despite several health insurance schemes majority Indian population continue to incur medicine OOP while seeking outpatient care.

The foregoing clearly points that several policy interventions and program design were conceived and implemented in the recent past to provide financial risk protection to households. However, gross underinvestment in public health system in past had led to inadequate prepayment and risk protection measures[24]. Several policy interventions and program redesign are required to reverse the trend of high OOP, which leads to catastrophe and impoverishment among significant sections of Indian population. For example, an efficient and a reliable supply chain model existed for over two and half decades in the state of Tamil Nadu, which was replicated in the state of Rajasthan in 2012 have been instrumental in improving access to medicines in the frontline facilities[32]. Such policies and

For peer review only - http://bmjopen.bmj.com/site/about/guidelines.xhtml

programs governing public health facilities are critical, what is even more vital is the price cap intervention in the retail segment. The NHP 2017 also highlighted the need for providing free medicines in public health facilities by stepping up funding and improving drug procurement and distribution mechanisms[33]. In addition to DPCO, 2013, the present central government has reduced prices of 200 key essential drugs whose prices were not notified by DPCO earlier[28], using a special provision under Para 19 of DPCO to bring more drugs under price ceiling. A recent pronouncement by the government intends to bring legislation for physicians to prescribe drugs only in generic names, holds even greater promise for reducing households' OOP. In summary, government intervention in terms of public provisioning of free medicine has potential to significantly reduce the medicine related OOP as well as total OOP.

Contributorship: AK and SS conceived the idea, designed the analysis, conducted data analysis and wrote the first draft of the paper. AK, SS and HHF conducted the literature review and the interpretation of the results. AS, SS and HHF revised and edited the manuscript to its final stages. All the authors approved the final manuscript version.

Acknowledgments: None

Competing Interests: We declare no conflict of interest.

Funding: Habib Hasan Farooqui is supported by a Welcome Trust Capacity Strengthening Strategic Award to the Public Health Foundation of India and a consortium of UK universities. The funders had no role in study design, data collection and analysis, decision to publish, or preparation of the manuscript.

Data sharing: The data used for the analysis is available in public domain.

Ethical approval: Ethical approval for this study was not needed. The study used only anonymised data from secondary sources. Requisite permission to use the data has been obtained from the agency.

Reference list:

1. (NHSRC). NHSRC. National Health Accounts Estimates for India 2013-14: Ministry of Health and Family Welfare (MoHFW). Government of India, 2014.
2. Van Doorslaer E ODO, Rannan-Eliya RP, Somanathan A, Adhikari SR, Garg CC, et al. . Effect of payments for health care on poverty estimates in 11 countries in Asia: an analysis of household survey data. *The Lancet* 2006;368:1357-64.
3. Doorslaer EV ODO, Rannan-Eliya RP, Somanathan A, Adhikari SR,, al. e. Catastrophic payments for health care in Asia. *Health Econ* 2007;16:1159–84.

For peer review only

4. Karan A, Selvaraj S, Mahal A. Moving to universal coverage? Trends in the burden of out-of-pocket payments for health care across social groups in India, 1999-2000 to 2011-12. *PloS one* 2014;9(8):e105162. doi: 10.1371/journal.pone.0105162
5. Berman P AR, Bhandari L. . The impoverishing effect of healthcare payments in India: New methodology and findings. . *Economic & Political Weekly* 2010;45:65-71.
6. Garg CC KA. Reducing out-of-pocket expenditures to reduce poverty: a disaggregated analysis at rural-urban and state level in India. . *Health Policy and planning ;* 2009; 24:116-28. .
7. Selvaraj S, Karan, A.K. Deepening health insecurity in India: evidence from national sample surveys since 1980s. . *Econ Political Wkly* 2009;44:55–60.
8. Wagstaff A. The economic consequences of health shocks: evidence from Vietnam. . *Health Econ* 2007. ;26 82–100.
9. Xu K, Evans, D.B., Kawabata, K., Zeramdini, R., Klavus, J., Murray, C.J.L. Household catastrophic health expenditure: a multicountry analysis. *Lancet* 2003;362 111–17.
10. Xu K, Evans, D.B., Carrin, G., Aguilar, A.M., Musgrove, P., Evans, T. Protecting households from catastrophic health spending. . *Health Aff* 2007;26(972–983.)
11. Ministry of Health and Family Welfare (MoHFW) GoI. NCMH: Report of the National Commission on Macroeconomics and Health. , 2005.
12. Rao KD, Bhatnagar A, Murphy A. Socio-economic inequalities in the financing of cardiovascular & diabetes inpatient treatment in India. *Indian J Med Res* 2011;133:57-63.
13. Khatib R, McKee M, Shannon H, et al. Availability and affordability of cardiovascular disease medicines and their effect on use in high-income, middle-income, and low-income countries: an analysis of the PURE study data. *Lancet* 2016;387(10013):61-9. doi: 10.1016/S0140-6736(15)00469-9
14. van Mourik MS, Cameron A, Ewen M, et al. Availability, price and affordability of cardiovascular medicines: a comparison across 36 countries using WHO/HAI data. *BMC Cardiovasc Disord* 2010;10:25. doi: 10.1186/1471-2261-10-25
15. Fan VY, Karan A, Mahal A. State health insurance and out-of-pocket health expenditures in Andhra Pradesh, India. *Int J Health Care Finance Econ* 2012;12(3):189-215. doi: 10.1007/s10754-012-9110-5
16. Commission. P. Report of the Expert Group to Review the Methodology for Measurement of Poverty. New Delhi: Planning Commission, Government of India. , 2014.
17. Bank W. World Bank 2017 [Available from: http://iresearch.worldbank.org/PovcalNet/FAQs.aspx; 2017.
18. Reddy Kea. High Level Expert Group (HLEG) report on Universal Health Coverage (UHC) for India. New Delhi: Public Health Foundation of India, 2011.
19. Selvaraj S, Chokshi, M., Hasan, H., & Kumar, P. . Improving Governance and Accountability in India’s Medicine Supply System. . New Delhi: Public Health Foundation of India., 2010.

For peer review only

20. Prinja S, Bahuguna P, Tripathy JP, et al. Availability of medicines in public sector health facilities of two North Indian States. *BMC Pharmacol Toxicol* 2015;16:43. doi: 10.1186/s40360-015-0043-8
21. Kotwani A, Gurbani N, Sharma S, et al. Insights for policymakers from a medicine price survey in Rajasthan. *Indian J Med Res* 2009;129(4):451-4.
22. Kotwani A. Where are we now: assessing the price, availability and affordability of essential medicines in Delhi as India plans free medicine for all. *BMC health services research* 2013;13:285. doi: 10.1186/1472-6963-13-285
23. Karan A, et al. Key indicators of morbidity, utilization and health expenditure - Tamil Nadu. New Dlehi: Public Health Foundation of India, 2017.
24. Mackintosh M, Channon A, Karan A, et al. What is the private sector? Understanding private provision in the health systems of low-income and middle-income countries. *Lancet* 2016;388(10044):596-605. doi: 10.1016/S0140-6736(16)00342-1
25. Selvaraj S KA. Deepening health insecurity in India: evidence from national sample surveys since 1980s. . *Econ Polit Wkly* 2009;44:55–60.
26. Kotwani A, Ewen M, Dey D, et al. Prices & availability of common medicines at six sites in India using a standard methodology. *Indian J Med Res* 2007;125(5):645-54.
27. India Go. Drug Price Control Order 1995. In: Department of Pharmaceuticals MoCaF, ed. India, 1995.
28. National Pharmaceutical Pricing Authority. Drug Price Control Order 2013. India, 2016.
29. Arvind Sahay SJ. Does Pharmaceutical Price Regulation Result in Greater Access to Essential Medicines? Study of the impact of drug price control order on sales volume of drugs in India. Ahmedabad: Indian Institute of Management, 2016.
30. Devadasan N, Seshadri, T., Trivedi, M., Criel, B. Promoting universal financial protection: evidence from the rashtriya Swasthya Bima Yojana (RSBY) in Gujarat, India. . *Health Res Policy Syst* 2013. ;11:1–29.
31. Prinja S, Chauhan AS, Karan A, et al. Impact of Publicly Financed Health Insurance Schemes on Healthcare Utilization and Financial Risk Protection in India: A Systematic Review. *PloS one* 2017;12(2):e0170996. doi: 10.1371/journal.pone.0170996
32. Singh PV, Tatambhotla A, Kalvakuntla R, et al. Understanding public drug procurement in India: a comparative qualitative study of five Indian states. *BMJ Open* 2013;3(2) doi: 10.1136/bmjopen-2012-001987
33. Ministry of Health and Family Welfare (MoHFW) GoI. NATIONAL HEALTH POLICY, 2017 2017 [Available from: https://www.nhp.gov.in//NHPfiles/national_health_policy_2017.pdf.

For peer review only

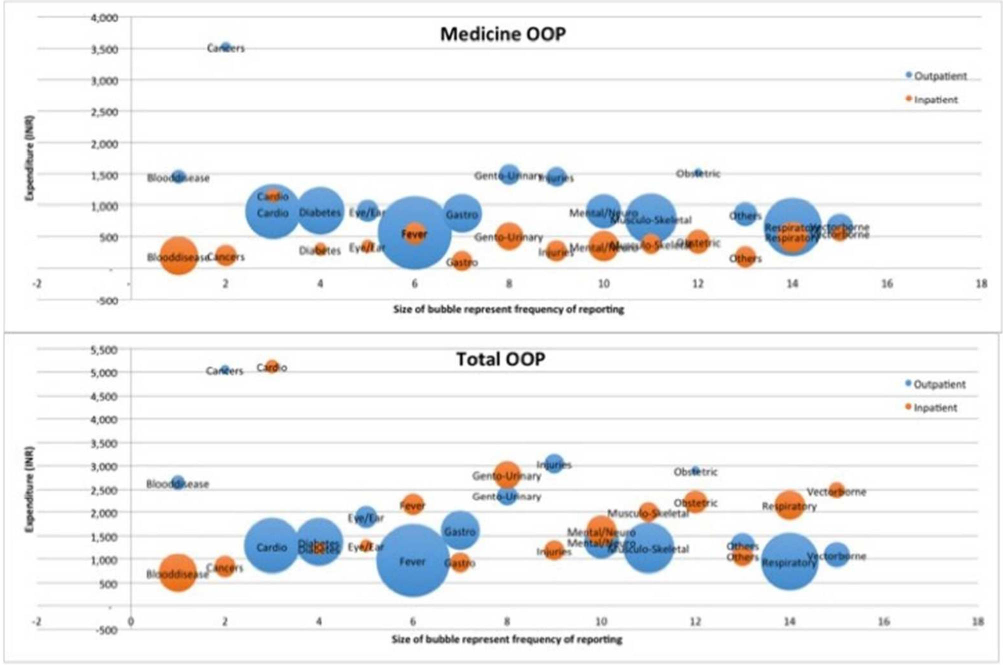

Figure 1: Disease wise average monthly per person medicine OOP and total OOP, 2014

255x178mm (72 x 72 DPI)

iew only

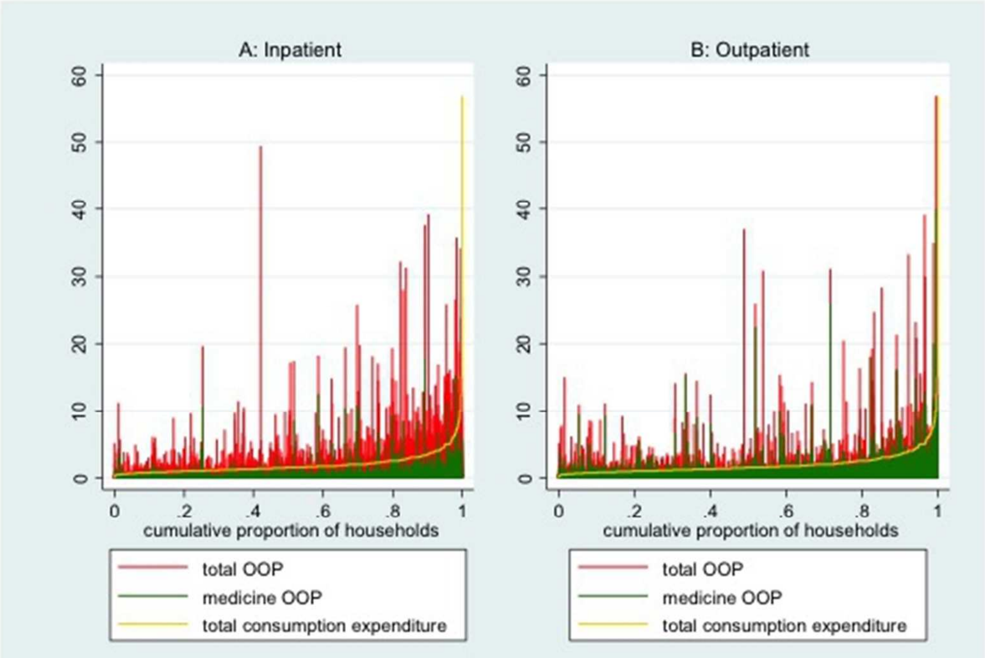

Figure2: Monthly per person total OOP; medicine OOP and consumption expenditure

200x146mm (72 x 72 DPI)

Supplementary materials

Annexure I: **Estimation of out-of-pocket expenditure indicators**

a. Out-of-pocket payments

Total out-of-pocket (OOP) payment has been defined as the summation of all kinds of direct expenditure on purchase of medical care including expenditure on family planning devices and transportation costs to access medical care by households either as inpatient or outpatient. According to the NSSO household questionnaire the main item of expenditure considered for inpatient and outpatient are presented in Table A-I.

Table A-I: Main items of expenditure considered for inpatient and outpatient in the NSSO questionnaire

**Inpatient**		**Outpatient**	
**Heads of expenditure**	**Item code in NSSO questionnaire**	**Heads of expenditure**	**Item code in NSSO questionnaire**
medicine	410	medicine	420
X-ray, ECG, pathological test, etc.	411	X-ray, ECG, pathological test, etc.	421
doctor’s/surgeon’s fee	412	doctor’s/ surgeon’s fee	422
hospital & nursing home charges	413	family planning devices	423
other medical expenses	414	other medical expenses	424
**Total inpatient**	**419=410-414**	**Total outpatient**	**429=420-424**

The reference period of inpatient and outpatient expenditure in the consumer expenditure surveys are 1 year and 1 month respectively. Based on the information presented in Table A-I, total inpatient expenditure, outpatient expenditure and total OOP expenditure were estimated by converting inpatient expenditure for one month. Accordingly, households with any OOP have been defined as households reporting positive OOP (OOP>0) either as inpatient or outpatient or both.

b. Per person monthly OOP

For peer review only

Per person monthly OOP is defined as total monthly OOP divided by household size for each household.

c. OOP share

The financial burden of health expenses by households has also been estimated in terms of OOP as a share of total household expenditure and alternatively as a share of total non-food expenditure of households..

$$Sh_{OOP} = T/\exp \quad \ldots\ldots\ldots\ldots\ldots\ldots\ldots\ldots \text{(A.1)}$$

Where, 'T' is total OOP payments and 'exp' is household total (non-food) expenditure by households.

d. Catastrophic payments and headcount

Further, OOP payments are defined as catastrophic when OOP payments as a portion of total household resources are in excess of a certain threshold. A household is said to have incurred catastrophic payments if T/exp>Z, where 'T' and OOP are the same as in equation (A.1) and 'Z' is a certain threshold. The latter is arbitrary and in general, estimates are presented for a range of values for z (5, 10, 15, 25 and 40 per cent).

Accordingly, the headcount ratio of catastrophic payment is calculated as follows:

$$cat_i = \frac{1}{n}\sum 1\left(\frac{T}{exp} > Z_i\right) \ldots\ldots\ldots\ldots\ldots\ldots\ldots\ldots\ldots\ldots\ldots\ldots \text{(A.2)}$$

Where, Cat_i is catastrophic headcounts of households with OOP share exceeding a threshold defined as 'i' per cent of total household total (non-food) expenditure, 1(.) is an indicator function, which takes the value 1 if T/exp>Zi is true and 0 otherwise; n is the number of households incurring expenditure on health for various thresholds; Z_1, Z_2, Z_3 …… are the respective thresholds of the OOP share.

d. Poverty headcount

The usual headcount ratio of poverty is calculated as:

$$pre\ HP = 1/n\ \Sigma\, 1\ (xi \leq PL) \ldots\ldots\ldots\ldots\ldots\ldots\ldots\ldots\ldots\ldots \text{(A.3)}$$

For peer review only

where: 1(.) is a function, taking the value 1 if person belong to a household with consumption expenditure lower the value of poverty line value, x= Household total consumption expenditure; PL= Poverty Line and n is total population.

Headcount of poverty after deducting OOP from household consumption expenditure can be defined as:

post HP = 1/n Σ 1 ((xi-T)≤ PL). ……………………………… (A.4)

where: T= per capita OOP

The OOP induced poverty headcount finally is estimated as: (*pre HP - post HP)*

Table 1. Average per episode total and medicine out of pocket payments by disease conditions in 2014

	Outpatient 15 days recall						Inpatient 365 days recall					
	Prevalance				estimated monthly		Prevalance				estimated monthly	
Ailment	Freq.	Percent	Total OOP	Drug	Total OOP	Drug	Freq.	Percent	Total OOP	Drug	Total OOP	Drug
Fever	2,71,43,431	22.71	488	280	975	561	74,12,043	12.95	8670	2329	713	191
TB/Filaria/Tetanus	11,08,425	0.93	524	287	1047	573	6,14,933	1.07	14731	4134	1,211	340
STD/HIV/AIDS	1,20,714	0.1	538	268	1076	536	88,935	0.16	6906	1633	568	134
Vectorborne	33,33,651	2.79	549	332	1097	663	22,65,189	3.96	10288	2460	846	202
Cancers	4,52,513	0.38	2527	1763	5054	3526	9,78,764	1.71	62297	14037	5,121	1,154
Blood disease	10,26,129	0.86	1322	731	2643	1463	8,10,752	1.42	15035	3650	1,236	300
Diabetes	1,17,55,081	9.84	683	456	1367	911	8,17,199	1.43	15746	4224	1,294	347
Other Metabolic	20,50,282	1.72	712	340	1423	679	2,60,707	0.46	15429	3600	1,268	296
Mental/Neuro	61,56,374	5.15	690	451	1380	902	24,87,836	4.35	26428	6685	2,172	549
Eye/Ear	24,67,286	2.06	950	454	1899	908	20,82,420	3.64	11350	1407	933	116
Cardio	1,55,65,223	13.02	645	449	1289	899	37,82,374	6.61	34167	6129	2,808	504
Respiratory	1,69,58,670	14.19	478	328	955	656	21,40,762	3.74	14491	3325	1,191	273
Gastro	77,13,330	6.45	809	434	1617	869	45,40,520	7.93	19587	4260	1,610	350

For peer review only

Skin	28,34,892	2.37	522	370	1043	740	3,80,346	0.66	12123	3514	996	289
Musculo-Skeletal	1,32,29,065	11.07	622	391	1244	782	19,66,211	3.44	24379	4677	2,004	384
Genito-Urinary	21,91,953	1.83	1183	747	2365	1494	28,01,133	4.89	27085	5094	2,226	419
Obstetric	3,64,060	0.3	1448	765	2896	1529	22,64,628	3.96	13050	2189	1,073	180
Injuries	19,93,646	1.67	1522	730	3045	1460	46,19,876	8.07	26242	6000	2,157	493
Others	28,92,298	2.42	655	426	1310	853	12,97,049	2.27	30196	6666	2,482	548
Childbirth	1,46,562	0.12	585	398	1169	797	1,56,17,000	27.29	8508	1729	699	142

For peer review only

BMJ Open

Quantifying the financial burden of households' out-of-pocket payments on medicines in India. A repeated cross-section analysis of National Sample Survey data 1994 to 2014

Journal:	*BMJ Open*
Manuscript ID	bmjopen-2017-018020.R1
Article Type:	Research
Date Submitted by the Author:	21-Nov-2017
Complete List of Authors:	Selvaraj, Sakthivel; Public Health Foundation of India,<br>Farooqui, Habib; Public Health Foundation of India,<br>Karan, Anup; Indian Institute of Public Health, Delhi (IIPHD), Public Health Foundation of India,
**Primary Subject Heading**:	Health economics
Secondary Subject Heading:	Health policy
Keywords:	HEALTH ECONOMICS, Health policy < HEALTH SERVICES ADMINISTRATION & MANAGEMENT, PUBLIC HEALTH

SCHOLARONE™
Manuscripts

For peer review only

Title page

Title of the article:

Quantifying the financial burden of households' out-of-pocket payments on medicines in India. A repeated cross-section analysis of National Sample Survey data 1994 to 2014

Full name, postal address, e-mail and telephone number of the corresponding author:

Dr. Habib Hasan Farooqui, MD
Associate Professor
Address: Public Health Foundation of India, Plot No. 47, Sector 44, Institutional Area, Gurugram, India -122001
Email: drhabibhasan@gmail.com
Phone: +91 9999984673

Full name, department, institution, city and country of all co-authors:

Dr. Sakthivel Selvaraj, PhD
Director,
Address: Public Health Foundation of India, Plot No. 47, Sector 44, Institutional Area, Gurugram, India -122001

Dr. Habib Hasan Farooqui, MD
Associate Professor
Address: Public Health Foundation of India, Plot No. 47, Sector 44, Institutional Area, Gurugram, India -122001

Dr. Anup Karan, DPhil
Associate Professor,
Address: Public Health Foundation of India, Plot No. 47, Sector 44, Institutional Area, Gurugram, India -122001

For peer review only

Quantifying the financial burden of households' out-of-pocket payments on medicines in India. A repeated cross-section analysis of National Sample Survey data 1994 to 2014

Objective: The objective of this research is to generate new evidence on financial implications of medicines out of pocket (OOP) payments for households. Another objective is to investigate which disease conditions contributed to a significant proportion of households' financial burden.

Setting: All Indian States including Union Territories, 1993-94 to 2014

Design: Repeated cross section household surveys

Data: Secondary data of nationwide Consumer Expenditure Surveys for the years 1993-94, 2004-05 and 2011-12 and one wave of Social Consumption: Health for the year 2014 from National Sample Survey Organisation.

Outcome measure : Out-of-pocket expenditure on healthcare in general and medicines in specific.

Results: Total OOP payments and medicines OOP payments were estimated to be 6.77% [CI: 6.70%, 6.84%] and 4.49% [4.45%, 4.54%] of total consumption expenditure respectively in the year 2011-12 which marked significant increase since 1993-94. These proportions were 11.46% [CI: 11.36%, 11.56%] and 7.60% [CI: 7.54%, 7.67%] of non-food expenditure respectively in the same year. It was observed that total OOP payments and medicines OOP payments were catastrophic for 17.9% [CI: 17.7%, 18.2%] and 11.2% [11.0%, 11.4%] households respectively in 2011-12 at the 10% of total consumption expenditure threshold, implying 29 million households incurred catastrophic out-of-pocket payments in the year 2011-12. Further, medicines out-of-pocket payments pushed 3.09% [CI: 2.99%, 3.20%], implying 38 million, persons into poverty in the year 2011-12. Among the leading cause of diseases that caused significant OOP payments are cancers, injuries, cardiovascular diseases, genito-urinary conditions and mental disorders.

Conclusions: Strengthening government intervention in providing medicines free in public health care facilities has the potential to considerably reduce medicines related spending as well as total OOP payments of households.

Key words: India, out of payment, medicine, catastrophic expenditure, and poverty

For peer review only

For peer review only - http://bmjopen.bmj.com/site/about/guidelines.xhtml

Article summary

Strengths and limitations of this study

1. The study used multiple points of time and nationally representative data set to highlight the financial burden of households OOP payments on medicines in India;
2. The paper links medicines out-of-pocket payments by households with leading disease conditions and identify key disease conditions which cause medicines out-of-pocket payments
3. The study has limitations as it uses arbitrary threshold for measuring catastrophic payments
4. The ailments, disease conditions and the associated OOP expenditure reported by the households in the survey are self-reports and not clinically diagnosed.

For peer review only

For peer review only - http://bmjopen.bmj.com/site/about/guidelines.xhtml

Background

Households' in India bear significant financial burden on account of medical treatment, as the current prepayment and risk pooling mechanisms are inadequate. Since both government funding and social health insurance contributions are insufficient to meet health care needs of households, over three-fourth of all health care payments are paid out-of-pocket (OOP) at the point of service delivery while medicines purchase (approximately 63%) account for the single largest component of these payments.[1] Available literature suggests that OOP spending has dominated total OOP payments over the years.[2] Hence, it can be suggested that expenditure on medicines is major cause of catastrophe at the household level.[3]

India has the distinction of being pharmacy of the global south - supplies affordable, life saving, quality generic medicines. It ranks 4th in terms of volumes and 13th in terms of value of pharmaceuticals produced globally.[4] However, according to a WHO report around 68% of the Indian population have limited or no access to essential medicines.[5] In addition, literature suggests that over last two decades availability of free medicines in public health facilities has declined from 31.2% to 8.9 % for inpatient care and from 17.8% to 5.9% for outpatient care.[6] Another study demonstrated that medicines purchase alone constituted over 70% of overall OOP payments. In addition, the study demonstrated that by removing OOP payments for outpatient care on medicines, the percentage of people falling below poverty because of spending on health reduced to just 0.5 % whereas removing OOP payments for inpatient care resulted in a negligible decline in poverty headcount ratio and poverty gap highlighting the role of medicines expenditure in healthcare related impoverishment.[7]

Utilising impoverishment tool to measure affordability, one study assessed the impoverishment effect of medicines purchases by households in 16 low-and-middle income economies.[8] Comparing four key medicine prices to household income, and using World Bank poverty levels of US$ 1.25 or US$ 2 per day, the study concluded that a substantial number of people had to bear financial burden due to unaffordability of medicines. For example, it was pointed out that an originator brand atenolol purchase by individuals would push an additional 22% of population below the US$ 1.25 per day measurement while even a generic equivalent of atenolol was likely to push about 7% of population below poverty levels in Philippines.[9] Analyzing economic implications of non-communicable disease in India, a few studies also reported in the past that households incur significant OOP payment burden in certain conditions like cardiovascular diseases (CVD) and cancers. [10,11] Using 2004 NSSO data, another study highlighted that hospitalization with CVD resulted in 12% higher odds of incurring catastrophic spending and 37% greater odds of falling into poverty. For cancer, the impact was greatest with the odds of catastrophic expenditures 170% higher than the odds of incurring catastrophic spending when hospital stays are due to a

For peer review only - http://bmjopen.bmj.com/site/about/guidelines.xhtml

communicable disease condition.[12] However these studies do not reflect on the relative contribution of medicine in total OOP burden for the diseases they analysed.

Available evidence, both global and Indian, provides insights about the incidence of catastrophic payments and impoverishment impact of rising households OOP payments.[2 3 13-15] The literature on equity dimensions involving both catastrophe and impoverishment has attempted to address complex methodological and statistical approaches in measurements. However, there is lack of evidence on catastrophe and impoverishment on account of household's medicine OOP expenditure not only from inpatient and outpatient treatment costs perspective but also from the disease specific dimension. Given that medicines contribute to more than 66.6% of out-of-pocket healthcare expenditure, the focus of this research is to explore the consequences of high medicines OOP spending at the household level. Further, we investigated which disease conditions are contributing to high financial burden on households. We attempted to answer - what is the relative burden of medicines OOP payments by households in total OOP payments, catastrophic and poverty headcounts? And which disease conditions cause a relatively higher financial disruption in the living status of households?

Materials and methods

Data

The study utilises secondary data from three waves of nationally representative 'Consumer Expenditure Surveys' (CES): 1993-94, 2004-05 and 2011-12, conducted by the National Sample Survey Organisation (NSSO). In addition, health and morbidity survey (HMS) 2014 of the NSSO was used for disease-wise distribution of OOP payment burden. While the sample size of CES varied between 100,000 to 125,000 thousand households across different rounds, the sample size in HMS 2014 was approximately 72,000 households.

The CES collect socioeconomic and demographic information on households with key focus is on household spending on roughly 350 food and non-food items. Out-of-pocket medical expenses incurred by households are separately recorded for inpatient and outpatient services. The recall periods are one-year and 30-days for inpatient and outpatient expenses respectively. HMS collects detailed information on morbidity pattern, utilisation of health care services and associated expenditure by households. The HMS too separately records expenditure for inpatient and outpatient. However, unlike in CES the recall period for outpatient in HMS is 15 days.

Both CES and HMS are repeated cross-section surveys that are representative at the national and state levels. All districts of a state are included for sampling purposes. Households in CES are sampled evenly in quarterly sub-rounds beginning on 1 July and ending on 30 June of the following year, with equal numbers of households allotted in each quarterly sub-round, to address seasonality. In HMS, survey was completed in two sub-

For peer review only - http://bmjopen.bmj.com/site/about/guidelines.xhtml

rounds during January1 to June 30, 2014. All estimates in the present paper are sample weighted.

Outcome indicators

Using CES, we estimated 4 household-level indicators involving financial burden of illness: i) per household member monthly OOP spending on medicines (inflation-adjusted), ii) OOP spending on medicines as a share of total household and non-food spending, and iii) percentage of households reporting catastrophic payments on medicines and iv) percentage of households slipping onto poverty after netting out medicines OOP payments from households' total consumption expenditure. Total OOP spending of households was estimated by adding together expenditure on components of OOP payments. For inpatient OOP payment, we considered institutional spending on medicines, X-ray, ECG, pathological tests etc., doctor's/surgeon's fee, hospital and nursing charges and other medical expenses. For outpatient OOP payment, the components of expenditure are medicines, X-ray, ECG, pathological tests etc., doctor's/surgeon's fee, family planning services and other medical expenses. Expenditure on medicines is directly reported in the data set, both for inpatient and outpatient services (see Table A-I in Annexure). All the analyses report mean OOP spending on two parallel tracks: aggregated (across components of OOP) OOP payments (henceforth referred to as 'total OOP') and OOP payments only on account of medicine purchase (henceforth referred to as medicine/drug OOP).

Catastrophic payment for health care is defined as OOP payments being higher to a pre-defined threshold of total household consumption expenditure or alternatively household's non-food expenditure. For measuring catastrophic expenditure,[2 9] instead of sticking to a particular threshold, we considered a range of thresholds.[2 16-18] We also considered alternative thresholds as OOP spending as a share of household non-food expenditure. For OOP payment induced poverty estimates, we used two different poverty lines: i) Indian official state-specific rural and urban poverty lines[19] and ii) international poverty line based on US $ 1 per day per person adjusted to US $ 1.9 purchasing power parity (PPP) per day per person for the year 2011-12[20]. Yet another important poverty indicator, which particularly estimates magnitude of poverty deepening, is poverty gap. Using both the poverty lines separately, we also estimated mean poverty gaps for the poor. Details of the method used for catastrophic and poverty estimates are presented in Annexure.

In addition, we used NSSO 2014 HMS data for estimating disease level total and medicine OOP spending separately for inpatient and outpatient. Unlike CES, OOP spending in NSSO 2014 has not been recorded as part of the total household consumption expenditure and instead of estimating disease-wise catastrophic headcount, we present distribution of disease conditions based on incidence of occurrence and range of OOP spending separately for outpatient (15 days recall converted for 30 days) and inpatient (365 days recall

For peer review only - http://bmjopen.bmj.com/site/about/guidelines.xhtml

converted for 30 days). This helped identifying disease conditions, separately for outpatient and inpatient, which are high frequency occurrence and greater incidence of OOP spending.

Results

First, we present basic financial burden indicators for the years 1993-94, 2004-05 and 2011-12 in Table 1. Over 80% of populations are reportedly spending out-of-pocket while seeking treatment, during 2011-12. The proportion of population reporting any OOP payments have increased sharply from about 60% during 1993-94 to 80% percent in 2011-12. In respect to medicines spending, approximately every OOP spending is associated with expenditure on medicines. There was a significant increase (more than 50%) in household's total consumption expenditure in real terms from INR 517 in 1993-84 to INR 794 in 2011-12. However, during the same period total OOP payments increased by more than 100% from INR 26 in 1993-94 to INR 54 in 2011-12 in real terms. The rise in OOP payments on medicines has been more than 70% during the same period. Consequently, the share of spending on health from households' overall consumption expenditure have registered sharp increase during the past two decades, from a moderate 4.8% during 1993-94 to nearly 7% in 2011-12. If we were to net out food expenditure from total household consumption spending, which are considered a necessity, the share of health spending remained stagnant but as high as 11-12% percent during the period under consideration. It may be observed that in 2011-12 medicines alone contributed up to 67% of the total OOP payments.

Table 1: **Financial burden indicators, India, 1993-94, 2004-05 and 2011-12**

**Financial burden indicators**	**1993-94**	**2004-05**	**2011-12**
**Percentage households reporting OOP payments**			
Total OOP payments (%)	59.2 [58.9, 59.5]	64.4 [64.2, 64.7]	80.5 [80.2, 80.7]
Medicines OOP payments (%)	57.5 [57.3, 57.8]	63.6 [63.3, 63.8]	79.0 [78.8, 79.3]
**Monthly per capita expenditure (INR at constant 1999-2000 prices*)**			
Total household consumption expenditure	517 [515, 519]	619 [616, 622]	794 [790, 799]
Total out-of-pocket expenditure on health	25.59 [24.61, 26.25]	36.3 [35.7, 37.0]	54.3 [53.3, 55.3]
Medicine out-of-pocket expenditure	20.86 [19.50, 21.25]	26.0 [25.6, 26.4]	36.1 [35.5, 36.8]
**Share of health to total household expenditure (%)**			
Share of total out-of-pocket expenditure to total household expenditure (%)	4.84 [4.78, 4.91]	5.78 [5.72, 5.83]	6.77 [6.70, 6.84]

For peer review only

Share of medicine out-of-pocket expenditure to total household expenditure (%)	3.93 [3.87, 3.98]	4.10 [4.06, 4.14]	4.49 [4.45, 4.54]
**Share of health to non-food household expenditure (%)**			
Share of total OOP payments to non-food expenditure (%)	12.37 [12.20, 12.55]	10.82 [10.72, 10.91]	11.46 [11.36, 11.56]
Share of medicines OOP payments to non-food expenditure (%)	10.02 [9.88, 10.17]	7.68 [7.62, 7.75]	7.60 [7.54, 7.67]

Notes: 1. numbers in brackets are 95% confidence interval; 2. State and rural-urban specific consumer price indices were used to convert current prices values at the constant 1999-2000 prices. 3. The current prices values for monthly per capita total OOP are 15.7, 41.8 and 111.2 and for medicine OOP are 12.8, 29.8 and 73.9 (all in INR) for the years 1993-94, 2004-05 and 2011-12 respectively.

A higher burden of households' OOP payment is often associated with impoverishment and catastrophe. In Table 2, we present a set of catastrophic cut-offs measured as a share of OOP payments to total household consumption expenditure and non-food expenditure. Estimates for both OOP payments as well as OOP payments underlying medicines expenditure by households are presented.

Table 2: Percentage of households incurring catastrophic payments with respect to total OOP spending and medicines OOP spending, 1993-94, 2004-05 and 2011-12

**Financial Health Equity Measurements**	1993-94 (%)	2004-05 (%)	2011-12 (%)	Estimated number of households (2011-12)
**Cut-off for catastrophe using total household expenditure**				
Total OOP payment > 5%	26.9 [26.6, 27.1]	28.7 [28.5, 30.0]	35.3 [35.0, 35.6]	9,01,07,225
Total OOP payment > 10%	13.9 [13.8, 14.2]	14.6 [14.4, 14.8]	17.9 [17.7, 18.2]	4,56,91,766
Total OOP payment > 25%	3.9 [3.8, 4.0]	3.5 [3.4, 3.6]	4.3 [4.2, 4.4]	1,09,76,234
Medicines OOP payment > 5%	23.3 [23.0, 23.5]	23.4 [23.2, 23.6]	27.0 [26.7, 27.2]	6,89,20,540
Medicines OOP payment > 10%	11.5 [11.3, 11.7]	10.2 [10.2, 10.4]	11.2 [11.0, 11.4]	2,85,89,261

For peer review only

Medicines OOP payment > 25%	02.9 [2.8, 2.9]	1.6 [1.5, 1.7]	1.8 [1.7, 1.9]	45,94,703
**Cut-off for catastrophe using non-food expenditure**				
Total OOP payment > 5%	47.8 [47.5, 48.1]	46.5 46.2, 46.8]	53.5 [53.2, 53.8]	13,65,64,775
Total OOP payment > 10%	34.8 [34.6, 35.1]	31.0 [30.7, 31.2]	34.9 [34.7, 35.2]	8,90,86,180
Total OOP payment > 25%	16.7 [16.5, 16.9]	11.4 [11.2, 11.5]	11.9 [11.7, 12.1]	3,03,76,090
Total OOP payment > 40%	9.7 [9.5, 9.9]	4.7 [4.6, 4.9]	4.9 [4.8, 5.0]	1,25,07,802
Medicines OOP payment > 5%	44.7 [44.4, 45.0]	42.5 [42.2, 42.8]	46.4 [46.1, 46.7]	11,84,41,225
Medicines OOP payment > 10%	31.2 [31.0, 31.5]	25.5 [25.3, 25.7]	26.1 25.9, 26.4]	6,66,23,189
Medicines OOP payment > 25%	13.9 [13.7, 14.1]	7.1 [7.0, 7.3]	6.3 [6.1, 6.4]	1,60,81,459
Medicines OOP payment > 40%	7.8 [7.6, 7.9]	2.2 [2.1, 2.3]	1.8 [1.7, 1.9]	45,94,703

Note: Figures in brackets are 95% confidence interval.

Over one-third of Indian households incurred OOP payments greater to 5% of total household expenditure in 2011-12. This percentage was lower in 1993-94 (27%) and 2004-05 (28%). At the 25% threshold of total household expenditure, over 4% households reported incurring OOP payments in 2011-12. This essentially translates to approximately 11 million Indian households . Out of these, more than 4.4 million households incurred such payments only on account of purchase of medicines. At a lower threshold of 10% of total household expenditure, the number of households facing catastrophe is approximately 46 millions, of which 29 million households incurred catastrophe on account of OOP payments on medicines alone. Considering only non-food expenditure of households as the basic living status variable, approximately similar number of households incurred medicines OOP payments in 2011-12 with OOP payments being as high as 40% of their non-food expenditure.

For peer review only

Table 3: Impoverishment Indicators due to households' total OOP and medicine spending , India, 1993-94, 2004-05 and 2011-12

	1993-94	2004-05	2011-12	Estimated population (2011-12)
**I. Using national poverty line***				
1. Headcount ratio indicators (%)				
Gross Headcount	45.32 [45.03, 45.61]	37.85 [37.58, 38.12]	22.17 [21.92, 22.43]	27,19,03,356
Headcount net of total OOP	49.52 [49.22, 49.81]	42.68 [42.40, 42.95]	26.65 [26.38, 26.92]	32,68,48,193
Total OOP payment induced poverty	4.20 [4.07, 4.30]	4.83 [4.71, 4.94]	4.48 [4.35, 4.60]	5,49,44,837
Headcount net of medicine OOP payment	48.91 [48.61, 49.20]	41.54 [41.27, 41.82]	25.27 [25.00, 25.53]	30,99,23,221
Medicine OOP payment induced poverty	3.59 [3.47, 3.69]	3.69 [3.59, 3.80]	3.09 [2.99, 3.20]	3,78,97,220
2. Poverty gap indicators (INR current prices)				
Gross poverty gap#	63.3 [62.9, 63.8]	103.4 [102.7, 104.2]	154.2 [152.3, 156.0]	
Gap net of total OOP payment ##	69.7 [69.3, 70.1]	115.8 [115.1, 116.5]	182.8 [181.0, 184.7]	
Total OOP payment induced gap##	6.4 [6.3, 6.5]	12.4 [12.2, 12.6]	28.6 [28.0, 29.2]	
Gap net of medicine OOP payment ###	68.9 [68.5, 69.3]	113.7 [113.0, 114.4]	176.7 [174.9, 178.5]	
Medicine OOP payment induced gap###	5.6 [5.5, 5.7]	10.3 [10.1, 10.4]	22.5 [22.0, 23.0]	

For peer review only

**II. Using international poverty line****				
1. Headcount ratio indicators (%)				
Gross Headcount	40.96 [40.67, 41.24]	33.07 [32.81, 33.34]	18.37 [18.13, 18.61]	22,52,98,360
Headcount net of total OOP payment	44.92 [44.63, 45.21]	37.38 [37.11, 37.65]	22.41 [22.16, 22.67]	27,48,46,829
Total OOP payment induced poverty	3.97 [3.85, 4.08]	4.31 [4.19, 4.42]	4.04 [3.92, 4.16]	4,95,48,469
Headcount net of medicine OOP payment	44.35 [44.06, 44.64]	36.34 [36.08, 36.61]	21.37 [21.11, 21.62]	26,20,91,778
Medicine OOP payment induced poverty	3.39 [3.29, 3.50]	3.27 [3.17, 3.68]	2.99 [2.89, 3.10]	3,66,70,773
2. Poverty gap indicators (INR current prices)				
Gross poverty gap#	59.3 [58.9, 59.7]	96.1 [95.3, 96.8]	150.7 [148.8, 152.7]	
Gap net of total OOP payment ##	65.4 [64.9, 65.8]	107.5 [106.8, 108.3]	177.0 [175.1, 179.1]	
Total OOP payment induced gap##	6.1 [6.0, 6.2)	11.5 [11.2, 11.7]	26.3 [25.7, 27.0]	
Gap net of medicine OOP payment ###	64.6 [64.2, 65.1]	105.8 [105.0, 106.5]	172.0 [170.0, 174.0]	
Medicine OOP payment induced gap###	5.3 [5.2, 5.4]	9.7 [9.5, 9.9]	21.3 [20.7, 21.8]	

Notes: based on Tendulkar Committee methods; ** using USD 1.90 PPP at 2011-12 prices and mixed recall period of household consumption expenditure; # only for poor; ## only for poor net of total OOP; ### only for poor net of medicine OOP

Next, we present implications of total and medicine OOP payments on poverty estimates (Table 3). To facilitate interpretation, we present 3 basic headcount ratio indicators: i) gross headcount – percentage of population below poverty line, ii) net of OOP headcount – percentage of population below poverty line after netting out OOP payments from

For peer review only

household consumption expenditure and iii) OOP payments induced poverty which is the difference of the first two reflecting rise in poverty ratio owing to OOP payments. The last two indicators are presented separately for total OOP payments and medicine OOP payments. Table 3 also provides estimates on poverty gap representing extent of poverty deepening in terms of monetary value. All these indicators are estimated using Indian official poverty line (Tendulkar Committee method) and international poverty line of US $ 1.90 PPP.

The difference in mean headcount measure of gross and net poverty ratios reflects the percentage of population falling below poverty line because of households' OOP payments on health care. The headcount ratio of households impoverished due to OOP payments was 3.97% during 1993-94, which inched up to 4.30% in 2004-05 while in 2011-12 it was at 4.04%, as per international poverty line. In terms of Indian state-specific official poverty lines, percentage of households falling below poverty line increased from 4.19% in 1993-94 to 4.48% in 2011-12. This translates to 55 million persons in 2011-12. Out of this, approximately 38 million became poor only because they had to purchase medicines through OOP payments. Using the same measurement, the headcount measure for households OOP payments on medicines appear to have marginally declined from 3.58% in 1993-94 to 3.09% during 2011-12 using the international poverty line. As far as poverty gap is concerned, based on the Indian official poverty line, total OOP payments and OOP payments on medicines resulted in poverty deepening among poor by INR 29 and INR 23 respectively in 2011-12. Further poverty deepening because of total and medicines OOP payments sharply increased in 2012 compared to that in the years 2004-05 and 1993-94.

OOP expenditure by disease conditions

We also conducted a disaggregated analysis on disease wise expenditure not only with reference to total OOP payments and medicines OOP payments but also by type of care - inpatient care versus outpatient care. The survey results suggested that most common health condition for seeking outpatient care was fever (22.7%) and for inpatient care was childbirth (27.3%). In addition, our estimates suggest that households incurred highest monthly per capita OOP spending both for inpatient and outpatient care on account of cancer treatment (INR 5,054 and INR 5,121 respectively) followed by injuries for outpatient care (INR 3,045) and cardiovascular events for inpatient care (INR 2,808).

We also mapped disease-wise expenditure, frequency of healthcare utilization and type of care to demonstrate that not only hospitalization but also outpatient care can lead to catastrophe and impoverishment of households (Figure 1). For example, our estimates suggest that monthly per capita medicines OOP payments for cancer care were significantly higher in outpatient care as compared to the inpatient care. However, as far as total OOP spending for cancer treatment is concerned, it is almost similar across inpatient and outpatient but significantly higher compared to that for other disease conditions. In

For peer review only - http://bmjopen.bmj.com/site/about/guidelines.xhtml

contrast, in respect to cardiovascular conditions, medicines OOP payments were similar for both inpatient and outpatient treatment but total OOP payments were significantly higher for inpatient treatment as against outpatient treatment. In treatments involving gastroenterology conditions, however, both medicines OOP payments and total OOP payments were higher for outpatient compared to inpatient treatment. Similarly, for mental disorders, medicines OOP payments were higher for outpatient care compared to inpatient but total OOP payments were almost similar both for outpatient and inpatient treatment. Therefore, it is noted that the average monthly medicines OOP payments were consistently higher for outpatient care as compared to inpatient care among key disease conditions. A relatively higher frequency of outpatient treatment visits compared to inpatient treatment coupled with a significantly larger medicines OOP payment may yield a higher incidence of catastrophe. A detailed estimate of prevalence and OOP payments by disease conditions cross-classified by inpatient and outpatient care are presented in Table A-II in Annexure.

Further, we plotted outpatient and inpatient OOP payments and medicines related OOP payments with respect to households 'usual' consumption expenditure. In Figure 2, households are ranked from the poorest to the richest on the X-axis based on their mean monthly per person consumption expenditure and on the Y-axis mean monthly per person OOP expenditure (total and medicine) are measured separately for outpatient and inpatient. It is observed that for a number of households, average monthly outpatient expenditure is not only significantly higher in relation to household's non-medical consumption expenditure but the frequency of such events is also higher in outpatient care as compared to inpatient care. In Figure 2, the concentration of red (total OOP payment) and green (medicine OOP payment) spikes above the consumption expenditure on the right hand side of the graph which reflects that even among richer households total OOP and medicine OOP payments are significantly higher than total non-medical consumption expenditure of households. Moreover, concentration of medicine OOP payments above households' non-medical consumption expenditure is more prominent in case of outpatient compare to the inpatient episodes.

Discussion and conclusion

Using standard methods of measuring catastrophe and impoverishment, [17 18] this paper demonstrates the financial burden of households' OOP payments on medicines in India, spanning two decades from 1993-94 to 2011-12. To our knowledge, this is a first attempt to link medicines' OOP spending to key diseases conditions. Two trends stand out clearly from our findings. First, the households' impoverishment on account of OOP expenditure is rather high and continued to be so during the last two decades. The impoverishment burden is largely driven by households spending on medicines, which accounted for over three-fourth of all medical impoverishment in India. Second, as far the catastrophe measurement is concerned, applying a 10% threshold of OOP payment on overall

For peer review only - http://bmjopen.bmj.com/site/about/guidelines.xhtml

consumption expenditure, an estimated 18% percent of Indian households appear to suffer financial catastrophe. Medicines' OOP expenditure alone contributed to an estimated 11% of financial catastrophe. In absolute numbers, this translates to a scenario where an estimated 46 million households appear to face catastrophic expenditure on account of OOP payments while 29 million households faced such hardship because they had to pay for medicines from their pockets.

Recent evidence from the National Health Accounts for India points out that during 2013-14, an estimated INR 1,331 per capita was spent on medicines, while households alone contributed INR 1,200 per capita, accounting for 90% of all medicines expenditure in the country.[1] On the other hand, past evidence about government expenditure on medicines in India, underscores that, on an average government spent about 10% percent of health expenditure on medicines. However, the national average masks significant underspending on medicines by several state governments, with many reportedly spending less than five percent of their health budgets.[6] Besides poor allocation of resources, except for a couple of Indian states, drug procurement and supply chain system is inefficient and ineffective leading to acute shortages of key essential medicines and chronic stock-outs in public health facilities.[21-24] This situation has resulted in physical unavailability of medicines. Drawing evidence from large sample surveys for the period from 1986-87 to 2004, it is reported the physical barrier to access to key essential medicines worsened during this period.[15] Supply of free drugs in government health system in the outpatient care setting, declined sharply from about 18% in 1986-87 to 5% in 2004. For the same period, drugs prescribed during hospitalization for free also declined significantly from one-third to about 9%.[6] As a result, it is pointed out the number of hospitalization episodes in which an ailing population paid out-of-pocket (OOP) payment, has risen dramatically from about 41% to close to 72%.[6] Further, it was observed that from the period spanning mid-1990s to 2004, patients visiting government health facilities did not receive medicines in over one-fourth of outpatient episodes. Affordability of medicines is an important access indicator, because it translates into poor access or no access for people who have low purchasing power.[3] The consumer behaviour theory also predicts that raising the price (via high OOP expenditure on medicines or high copayment) for a service in the public health sector will move more consumers into the private sector, depending on the elasticity of substitution and transaction costs in the public sector.[25]

In view of inadequate availability of medicines in government health facilities, households end up accessing private facilities where they end up incurring significant OOP payment, in the absence of any financial risk protection. Past evidence suggests that the trend has sharpened in the last couple of decades. For instance, the percentage of population accessing private facilities for inpatient and outpatient treatment has accelerated significantly between 1986-87 and 2004. It may be observed that households accessing private hospital for inpatient care increased from around 40% to nearly 60% in rural India

For peer review only - http://bmjopen.bmj.com/site/about/guidelines.xhtml

while urban India reported a rise from 40% to 68%.[26] During the same period, outpatient care visits in private facilities remained high at around 75% in 1986-87 in rural India and 73% in urban India stepped up to 78% and 80% respectively for rural and urban India.[3]

The other critical evidence emerging from this paper focuses on disease specific medicines expenditure. The results demonstrate a pattern where households' medicine spending is concentrated on low frequency, high-value spending and high frequency, high-value spending. By disease-wise classification, expenditure on treatment of cancers, CVDs and injuries, both for outpatient and inpatient care dominate the spending pattern. Available literature confirms such an expenditure pattern, wherein the share of non-communicable diseases (cardiovascular disease, diabetes, cancer, mental illness, injuries and others) in OOP health expenses has increased from 31.6% in 1995-96 to 47.3% in 2004.[12] The literature further indicates high odds of catastrophic hospitalization expenditures for certain NCDs. For example, the odds for catastrophic expenditure in cancer are nearly 170% greater, for cardiovascular diseases (CVD) and injuries nearly 22% greater than the odds due to infectious diseases. Other studies on cardiovascular diseases highlighted that CVD affected households had more outpatient visits and inpatient stays, spent extra money per hospitalization [11] and have high probability of incurring catastrophic expenditure.[27] Another Indian study on socio-economic inequalities in financing of diabetes and cardiovascular disease reported that out-of-pocket payments for hospital treatment claimed a large share of annual household expenditures; 30% for CVD and 17% for diabetes.[28] In respect to injuries (both road traffic and non-road traffic), high incidence of catastrophic expenditure was 30%, and was significantly higher among those belonging to the lowest income quartile and with an inpatient stay greater than 7 days.[29]

Although public facilities have slightly stepped up their share in outpatient care in recent years, private sector continue to dominate both in outpatient and inpatient care in India.[30] As an increasing share of households' access private health facilities, private retail pharmacies have become a major source of supply of key essential medicines. While availability of medicines may per se is not a challenge in the private health care setting, affordability appears to act as critical barrier.[31] Thus, pricing of medicines and regulation around retail medicine prices becomes a key factor in improving affordability and thereby leading to a reduction in medicines related OOP payment burden. Although India had a progressive retail price cap policies since 1979, but over the years a policy of deregulation was followed.[32] In 2013, the Government of India promulgated the Drugs Price Control Order (DPCO), 2013 (DPCO, 2013) which primarily brought all essential drugs, based on National List of Essential Medicines, 2011, under price control.[33] An evaluation of new price regulation has highlighted that, while few of the medicines (37) had an increase in sales volume attributable to DPCO, majority of the medicines (52) had a negative impact on their sales volume due to DPCO. Overall, the DPCO may have had a negative impact in terms of sales volume of medicines under price control.[34] Given that the sales volume of price-

For peer review only

capped medicines has declined, households OOP spending may continue to increase since over 80% of retail pharmacy market is not price-capped.

In order to improve access to health care and to provide financial risk protection to households, the central government and several state governments have been implementing a publicly funded health insurance programs since 2007, whose primary aim was to provide cashless treatment to economically vulnerable households for hospitalization episodes. Emerging evidence from micro as well as macro level studies point to a trend where such insurance schemes appear to have improved access to hospital care but have been ineffective in preventing financial catastrophe and impoverishment to households.[2 35 36] These bodies of evidence are in line with our findings that hospitalization based treatment cost constitute only one-third of India's morbidity burden. Despite implementation of several health insurance schemes, a majority of Indian population continues to incur a relatively significant medicines OOP payment while seeking outpatient care. It would be pertinent to highlight that the frequency of hospitalization is considerably smaller than outpatient visits in general, especially for non-communicable diseases (NCDs), which are chronic in nature that require multiple consultations and long-term or life-long medication support. Such medical conditions result in catastrophic expenditure for households even in the absence of hospitalization episodes. Moreover, since a relatively larger proportion of population seeks outpatient care in private facilities, which is often multiple times expensive than public health facilities, we observe a disproportionately higher burden of medicines related OOP payment for outpatient care.

The evidence presented in this paper, however, suffers from a few limitations. The first set of challenge relates to co-morbidities and associated expenditure. In respect to inpatient cases, since NSSO data captures disease expenditure separately for various disease conditions, the issue of co-morbid conditions did not play major role. However, for outpatient cases, we had to adopt apportioning technique to handle co-morbid conditions. The second set of challenge pertains to the potential recall bias for disease specific expenditures, which cannot be ruled out especially for hospitalization treatment since the recall period is a longer time span of 365 days.

The foregoing underlines several policy interventions and program design that were conceived and implemented in the recent past to provide financial risk protection to households. However, gross underinvestment in the public health system in past had led to inadequate prepayment and risk pooling measures.[26] Several policy interventions and program redesign are required to reverse the trend of high OOP expenditure for healthcare in India. An efficient and a reliable medicines supply chain model existed for over two and half decades in the state of Tamil Nadu, which was replicated in the state of Rajasthan in 2012 have been instrumental in improving access to medicines in the frontline facilities in these two states.[37] Such policies and programs governing public health facilities are critical.

For peer review only - http://bmjopen.bmj.com/site/about/guidelines.xhtml

The National Health Policy 2017 also highlighted the need for providing free medicines in public health facilities by stepping up funding and improving drug procurement and supply chain mechanisms.[38] A recent pronouncement by the government intends to bring legislation for physicians to prescribe drugs only in generic names, holds even greater promise for reducing households' OOP payments on medicines and ultimately providing financial risk protection. To sum up, both national and state governments' intervention is required for providing free medicines in public health facilities along with expanding the mechanism of price capping of key essential medicines in the private market.

Contributorship: AK and SS conceived the idea, designed the analysis, conducted data analysis and wrote the first draft of the paper. AK, SS and HHF conducted the literature review and the interpretation of the results. AS, SS and HHF revised and edited the manuscript to its final stages. All the authors approved the final manuscript version.

Acknowledgments: None

Competing Interests: We declare no conflict of interest.

Funding: Habib Hasan Farooqui is supported by a Welcome Trust Capacity Strengthening Strategic Award to the Public Health Foundation of India and a consortium of UK universities. The funders had no role in study design, data collection and analysis, decision to publish, or preparation of the manuscript.

Data sharing: The data used for the analysis is available in public domain.

Ethical approval: Ethical approval for this study was not needed. The study used only anonymised data from secondary sources. Requisite permission to use the data has been obtained from the agency.

Reference list:

1. (NHSRC). NHSRC. National Health Accounts Estimates for India 2013-14: Ministry of Health and Family Welfare (MoHFW). Government of India, 2014.
2. Karan A, Selvaraj S, Mahal A. Moving to universal coverage? Trends in the burden of out-of-pocket payments for health care across social groups in India, 1999-2000 to 2011-12. *PloS one* 2014;9(8):e105162. doi: 10.1371/journal.pone.0105162
3. Selvaraj S, Karan A. Deepening health insecurity in India: evidence from national sample surveys since 1980s. . *Econ Polit Wkly* 2009;44:55–60.
4. WHO. The World Medicines Situation 2011. Geneva: World Health Organozation, 2011.
5. WHO. The World Medicine Situation 2004. Geneva: World Health Organization, 2004.
6. Reddy K. High Level Expert Group (HLEG) report on Universal Health Coverage (UHC) for India. New Delhi: Public Health Foundation of India, 2011.

For peer review only

7. Shahrawat R, Rao KD. Insured yet vulnerable: out-of-pocket payments and India's poor. *Health policy and planning* 2012;27(3):213-21. doi: 10.1093/heapol/czr029
8. Khatib R, McKee M, Shannon H, et al. Availability and affordability of cardiovascular disease medicines and their effect on use in high-income, middle-income, and low-income countries: an analysis of the PURE study data. *Lancet* 2016;387(10013):61-9. doi: 10.1016/S0140-6736(15)00469-9
9. van Mourik MS, Cameron A, Ewen M, et al. Availability, price and affordability of cardiovascular medicines: a comparison across 36 countries using WHO/HAI data. *BMC Cardiovasc Disord* 2010;10:25. doi: 10.1186/1471-2261-10-25
10. Mahal A, Karan A, Fan VY, et al. The economic burden of cancers on Indian households. *PloS one* 2013;8(8):e71853. doi: 10.1371/journal.pone.0071853
11. Karan A, Engelgau M, Mahal A. The household-level economic burden of heart disease in India. *Trop Med Int Health* 2014;19(5):581-91. doi: 10.1111/tmi.12281
12. Engelgau MM, Karan A, Mahal A. The Economic impact of Non-communicable Diseases on households in India. *Globalization and health* 2012;8:9. doi: 10.1186/1744-8603-8-9
13. Khan JAM, Ahmed S, Evans TG. Catastrophic healthcare expenditure and poverty related to out-of-pocket payments for healthcare in Bangladesh-an estimation of financial risk protection of universal health coverage. *Health policy and planning* 2017;32(8):1102-10. doi: 10.1093/heapol/czx048
14. Ghosh S. Catastrophic Payments and Impoverishment due to Out-of-Pocket Health Spending. *Econ Political Wkly* 2011;46(47)
15. Selvaraj S, Karan A. Deepening health insecurity in India: evidence from national sample surveys since 1980s. . *Econ Political Wkly* 2009;44:55–60.
16. Fan VY, Karan A, Mahal A. State health insurance and out-of-pocket health expenditures in Andhra Pradesh, India. *Int J Health Care Finance Econ* 2012;12(3):189-215. doi: 10.1007/s10754-012-9110-5
17. Van Doorslaer E ODO, Rannan-Eliya RP, Somanathan A, Adhikari SR, Garg CC, et al. . Effect of payments for health care on poverty estimates in 11 countries in Asia: an analysis of household survey data. *The Lancet* 2006;368:1357-64.
18. Xu K, Evans, D.B., Kawabata, K., Zeramdini, R., Klavus, J., Murray, C.J.L. Household catastrophic health expenditure: a multicountry analysis. *Lancet* 2003;362 111–17.
19. GOI. Report of the Expert Group to Review the Methodology for Measurement of Poverty. New Delhi.: Planning Commission., 2014.
20. Bank W. World Bank 2017 [Available from: http://iresearch.worldbank.org/PovcalNet/FAQs.aspx; 2017.
21. Selvaraj S, Chokshi, M., Hasan, H., & Kumar, P. . Improving Governance and Accountability in India’s Medicine Supply System. . New Delhi: Public Health Foundation of India., 2010.
22. Prinja S, Bahuguna P, Tripathy JP, et al. Availability of medicines in public sector health facilities of two North Indian States. *BMC Pharmacol Toxicol* 2015;16:43. doi: 10.1186/s40360-015-0043-8
23. Kotwani A, Gurbani N, Sharma S, et al. Insights for policymakers from a medicine price survey in Rajasthan. *Indian J Med Res* 2009;129(4):451-4.
24. Kotwani A. Where are we now: assessing the price, availability and affordability of essential medicines in Delhi as India plans free medicine for all. *BMC health services research* 2013;13:285. doi: 10.1186/1472-6963-13-285

For peer review only

25. Alsan M SL, Eggleston K, Kammili N, Kolli P, Bhattacharya J. . Out-of-pocket health expenditures and antimicrobial resistance in low-income and middle-income countries: an economic analysis. . *Lancet Infect Dis* 2015;15(10):(10):1203-10.
26. Mackintosh M, Channon A, Karan A, et al. What is the private sector? Understanding private provision in the health systems of low-income and middle-income countries. *Lancet* 2016;388(10044):596-605. doi: 10.1016/S0140-6736(16)00342-1
27. Huffman MD, Rao KD, Pichon-Riviere A, et al. A cross-sectional study of the microeconomic impact of cardiovascular disease hospitalization in four low- and middle-income countries. *PloS one* 2011;6(6):e20821. doi: 10.1371/journal.pone.0020821
28. Rao K, Bhatnagar A, Murphy A. Socio-economic inequalities in the financing of cardiovascular & diabetes inpatient treatment in India. *Indian J Med Res* 2011;133:57-63.
29. Prinja S, Jagnoor J, Chauhan A, et al. Economic Burden of Hospitalization Due to Injuries in North India: A Cohort Study. *Int J Environ Res Public Health* 2016;13:673.
30. Karan A. Key indicators of morbidity, utilization and health expenditure - Tamil Nadu. New Dlehi: Public Health Foundation of India, 2017.
31. Kotwani A, Ewen M, Dey D, et al. Prices & availability of common medicines at six sites in India using a standard methodology. *Indian J Med Res* 2007;125(5):645-54.
32. GOI. Drug Price Control Order 1995. In: Pharmaceuticals. Do, ed. India, 1995.
33. NPPA. Drug Price Control Order 2013. India, 2016.
34. Sahay A, Jaikumar S. Does Pharmaceutical Price Regulation Result in Greater Access to Essential Medicines? Study of the impact of drug price control order on sales volume of drugs in India. Ahmedabad.: Indian Institute of Management., 2016.
35. Devadasan N, Seshadri, T., Trivedi, M., Criel, B. Promoting universal financial protection: evidence from the rashtriya Swasthya Bima Yojana (RSBY) in Gujarat, India. . *Health Res Policy Syst* 2013. ;11:1–29.
36. Prinja S, Chauhan AS, Karan A, et al. Impact of Publicly Financed Health Insurance Schemes on Healthcare Utilization and Financial Risk Protection in India: A Systematic Review. *PloS one* 2017;12(2):e0170996. doi: 10.1371/journal.pone.0170996
37. Singh PV, Tatambhotla A, Kalvakuntla R, et al. Understanding public drug procurement in India: a comparative qualitative study of five Indian states. *BMJ Open* 2013;3(2) doi: 10.1136/bmjopen-2012-001987
38. Ministry of Health and Family Welfare (MoHFW) GoI. NATIONAL HEALTH POLICY, 2017 2017 [Available from: https://www.nhp.gov.in//NHPfiles/national_health_policy_2017.pdf.

Figure legend:

Figure 1: Frequency and monthly per person (a) total OOP and (b) medicine OOP spending on select disease conditions, 2014

Figure 2: Monthly per person total OOP payment, medicine OOP payment and consumption expenditure

For peer review only

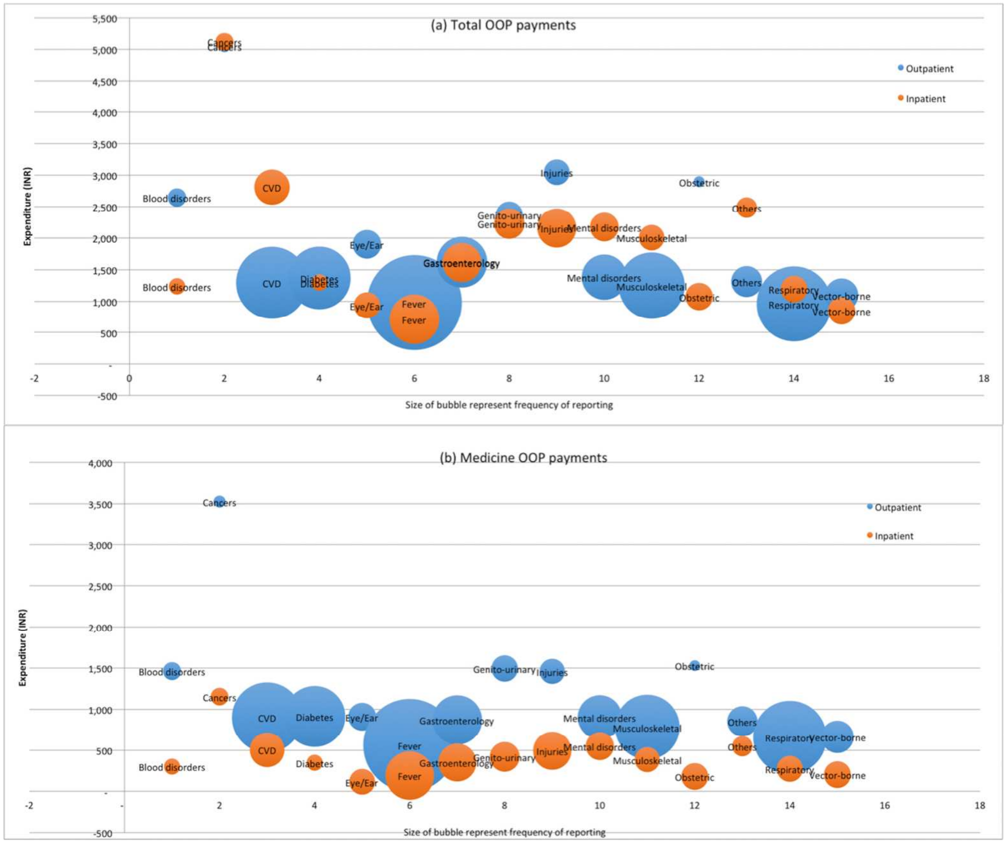

Frequency and monthly per person (a) total OOP and (b) medicine OOP spending on select disease conditions, 2014

228x190mm (300 x 300 DPI)

For peer review only - http://bmjopen.bmj.com/site/about/guidelines.xhtml

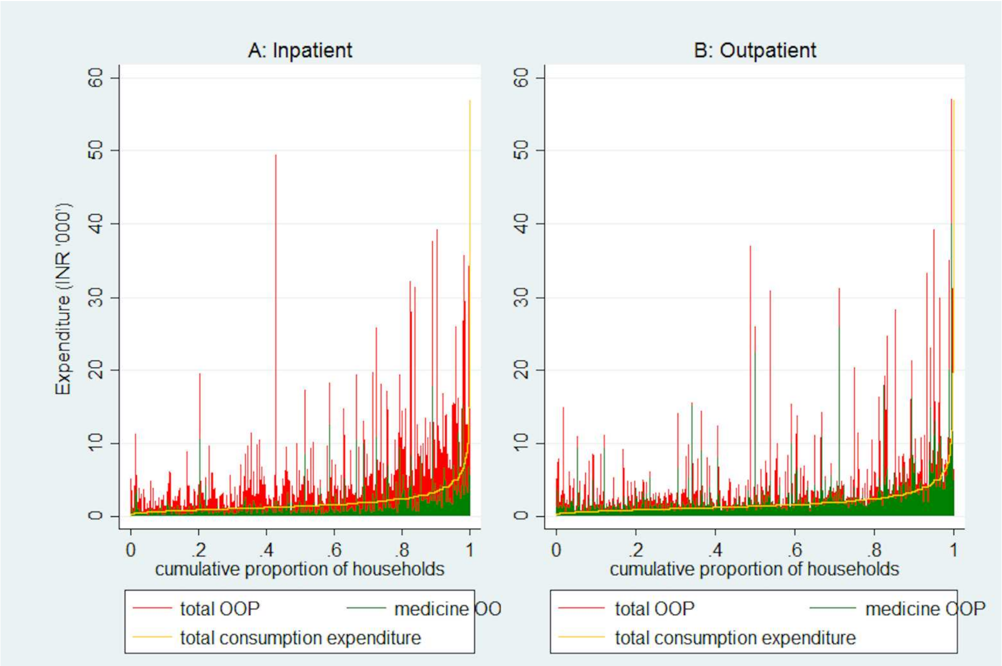

Figure2: Monthly per person total OOP; medicine OOP and consumption expenditure

Monthly per person total OOP payment, medicine OOP payment and consumption expenditure

302x219mm (300 x 300 DPI)

ew only

Annexure

Estimation of out-of-pocket expenditure indicators and related indicators

a. Out-of-pocket payments

Total out-of-pocket (OOP) payment has been defined as the summation of all kinds of direct expenditure on purchase of medical care including expenditure on family planning devices and transportation costs to access medical care by households either as inpatient or outpatient. According to the NSSO household questionnaire the main item of expenditure considered for inpatient and outpatient are presented in Table A-I.

Table A-I: Main items of expenditure considered for inpatient and outpatient in the NSSO questionnaire

**Inpatient**		**Outpatient**	
**Heads of expenditure**	**Item code in NSSO questionnaire**	**Heads of expenditure**	**Item code in NSSO questionnaire**
medicine	410	medicine	420
X-ray, ECG, pathological test, etc.	411	X-ray, ECG, pathological test, etc.	421
doctor's/surgeon's fee	412	doctor's/ surgeon's fee	422
hospital & nursing home charges	413	family planning devices	423
other medical expenses	414	other medical expenses	424
**Total inpatient**	**419=410-414**	**Total outpatient**	**429=420-424**

The reference period of inpatient and outpatient expenditure in the consumer expenditure surveys are 1 year and 1 month respectively. Based on the information presented in Table A-I, total inpatient expenditure, outpatient expenditure and total OOP expenditure were estimated by converting inpatient expenditure for one month. Accordingly, households with any OOP have been defined as households reporting positive OOP (OOP>0) either as inpatient or outpatient or both.

b. Per person monthly OOP and OOP share

For peer review only - http://bmjopen.bmj.com/site/about/guidelines.xhtml

Per person monthly OOP is defined as total monthly OOP divided by household size for each household.

The financial burden of health expenses by households has also been estimated in terms of OOP as a share of total household expenditure and alternatively as a share of total non-food expenditure of households..

$$Sh_{OOP} = T/\exp \quad \ldots\ldots\ldots\ldots\ldots\ldots\ldots\ldots \text{(A.1)}$$

Where, 'T' is total OOP payments and 'exp' is household total (non-food) expenditure by households.

c. Catastrophic payments and headcount

Further, OOP payments are defined as catastrophic when OOP payments as a portion of total household resources are in excess of a certain threshold. A household is said to have incurred catastrophic payments if T/exp>Z, where 'T' and OOP are the same as in equation (A.1) and 'Z' is a certain threshold. The latter is arbitrary and in general, estimates are presented for a range of values for z (5, 10, 15, 25 and 40 per cent).

Accordingly, the headcount ratio of catastrophic payment is calculated as follows:

$$cat_i = \frac{1}{n}\sum 1\left(\frac{T}{exp} > Z_i\right) \ldots\ldots\ldots\ldots\ldots\ldots\ldots\ldots\ldots\ldots\ldots\ldots \text{(A.2)}$$

Where, Cat_i is catastrophic headcounts of households with OOP share exceeding a threshold defined as 'i' per cent of total household total (non-food) expenditure, 1(.) is an indicator function, which takes the value 1 if T/exp>Zi is true and 0 otherwise; n is the number of households incurring expenditure on health for various thresholds; Z_1, Z_2, Z_3 …… are the respective thresholds of the OOP share.

d. Poverty headcount

The usual headcount ratio of poverty is calculated as:

$$Gross\ HP = 1/n\ \Sigma 1\ (x_i \leq PL) \ldots\ldots\ldots\ldots\ldots\ldots\ldots\ldots\ldots\ldots \text{(A.3)}$$

where: 1(.) is a function, taking the value 1 if person belong to a household with consumption expenditure lower the value of poverty line value, x= Household total consumption expenditure; PL= Poverty Line and n is total population.

Headcount of poverty after deducting OOP from household consumption expenditure can be defined as:

For peer review only

Net HP = 1/n Σ 1 ((xi-T)≤ PL). ………………………………. (A.4)

where: T= per capita OOP

The OOP induced poverty headcount finally is estimated as: (*Gross HP - Net HP*).

e. Poverty gap

Poverty gap is defined as difference between values of poverty lines and household consumption expenditure for the poor as defined in the sub-section d above. Using the household level data poverty gap for the poor is estimated as follows:

Gross poverty gap = 1/n Σ 1 (xi- PL)| if i=poor …………………………… (A.5)

In equation A.5 'xi-PL' is the difference between household expenditure per person and poverty line. For all positive values of this difference mean gap is estimated. Finally, poverty gap net of OOP payments is estimated as follows:

Net poverty gap = 1/n Σ 1 ((xi-T)- PL)| if i=poor after netting OOP ……… (A.6)

Table A-II. Prevalence and average per episode total and medicine out of pocket payments by disease conditions in 2014

	Outpatient 15 days recall						Inpatient 365 days recall					
	Prevalence		Per episode		estimated monthly		Prevalence		Per episode		estimated monthly	
Ailment	Number of episodes	%	Total OOP	Drug	Total OOP	Drug	Number of episodes	%	Total OOP	Drug	Total OOP	Drug
Fever	2,71,43,431	22.71	488	280	975	561	74,12,043	12.95	8670	2329	713	191
TB/Filaria/Tetanus	11,08,425	0.93	524	287	1047	573	6,14,933	1.07	14731	4134	1,211	340
STD/HIV/AIDS	1,20,714	0.1	538	268	1076	536	88,935	0.16	6906	1633	568	134
Vector-borne	33,33,651	2.79	549	332	1097	663	22,65,189	3.96	10288	2460	846	202

For peer review only - http://bmjopen.bmj.com/site/about/guidelines.xhtml

Cancers	4,52,513	0.38	2527	1763	5054	3526	9,78,764	1.71	62297	14037	5,121	1,154
Blood disease	10,26,129	0.86	1322	731	2643	1463	8,10,752	1.42	15035	3650	1,236	300
Diabetes	1,17,55,081	9.84	683	456	1367	911	8,17,199	1.43	15746	4224	1,294	347
Other Metabolic	20,50,282	1.72	712	340	1423	679	2,60,707	0.46	15429	3600	1,268	296
Mental disorders	61,56,374	5.15	690	451	1380	902	24,87,836	4.35	26428	6685	2,172	549
Eye/Ear	24,67,286	2.06	950	454	1899	908	20,82,420	3.64	11350	1407	933	116
Cardiovascular diseases	1,55,65,223	13.02	645	449	1289	899	37,82,374	6.61	34167	6129	2,808	504
Respiratory diseases	1,69,58,670	14.19	478	328	955	656	21,40,762	3.74	14491	3325	1,191	273
Gastroenterlogy	77,13,330	6.45	809	434	1617	869	45,40,520	7.93	19587	4260	1,610	350
Skin	28,34,892	2.37	522	370	1043	740	3,80,346	0.66	12123	3514	996	289
Musculo-Skeletal	1,32,29,065	11.07	622	391	1244	782	19,66,211	3.44	24379	4677	2,004	384
Genito-Urinary	21,91,953	1.83	1183	747	2365	1494	28,01,133	4.89	27085	5094	2,226	419
Obstetric	3,64,060	0.3	1448	765	2896	1529	22,64,628	3.96	13050	2189	1,073	180
Injuries	19,93,646	1.67	1522	730	3045	1460	46,19,876	8.07	26242	6000	2,157	493
Others	28,92,298	2.42	655	426	1310	853	12,97,049	2.27	30196	6666	2,482	548
Childbirth	1,46,562	0.12	585	398	1169	797	1,56,17,000	27.29	8508	1729	699	142

For peer review only - http://bmjopen.bmj.com/site/about/guidelines.xhtml

BMJ Open

Quantifying the financial burden of households' out-of-pocket payments on medicines in India. A repeated cross-section analysis of National Sample Survey data 1994 to 2014

Journal:	*BMJ Open*
Manuscript ID	bmjopen-2017-018020.R2
Article Type:	Research
Date Submitted by the Author:	18-Jan-2018
Complete List of Authors:	Selvaraj, Sakthivel; Public Health Foundation of India,<br>Farooqui, Habib; Public Health Foundation of India,<br>Karan, Anup; Indian Institute of Public Health, Delhi (IIPHD), Public Health Foundation of India,
**Primary Subject Heading**:	Health economics
Secondary Subject Heading:	Health policy
Keywords:	HEALTH ECONOMICS, Health policy < HEALTH SERVICES ADMINISTRATION & MANAGEMENT, PUBLIC HEALTH

SCHOLARONE™ Manuscripts

For peer review only

Title page

Title of the article:

Quantifying the financial burden of households' out-of-pocket payments on medicines in India. A repeated cross-section analysis of National Sample Survey data 1994 to 2014

Full name, postal address, e-mail and telephone number of the corresponding author:

Dr. Habib Hasan Farooqui, MD
Associate Professor
Address: Public Health Foundation of India, Plot No. 47, Sector 44, Institutional Area, Gurugram, India -122001
Email: drhabibhasan@gmail.com
Phone: +91 9999984673

Full name, department, institution, city and country of all co-authors:

Dr. Sakthivel Selvaraj, PhD
Director,
Address: Public Health Foundation of India, Plot No. 47, Sector 44, Institutional Area, Gurugram, India -122001

Dr. Habib Hasan Farooqui, MD
Associate Professor
Address: Public Health Foundation of India, Plot No. 47, Sector 44, Institutional Area, Gurugram, India -122001

Dr. Anup Karan, DPhil
Associate Professor,
Address: Public Health Foundation of India, Plot No. 47, Sector 44, Institutional Area, Gurugram, India -122001

For peer review only

Quantifying the financial burden of households' out-of-pocket payments on medicines in India. A repeated cross-section analysis of National Sample Survey data 1994 to 2014

Objective: The objective of this research is to generate new evidence on financial implications of medicines out of pocket (OOP) payments for households. Another objective is to investigate which disease conditions contributed to a significant proportion of households' financial burden.

Setting: All Indian States including Union Territories, 1993-94 to 2014

Design: Repeated cross section household surveys

Data: Secondary data of nationwide Consumer Expenditure Surveys for the years 1993-94, 2004-05 and 2011-12 and one wave of Social Consumption: Health for the year 2014 from National Sample Survey Organisation.

Outcome measures: Out-of-pocket expenditure on healthcare in general and medicines in specific.

Results: Total OOP payments and medicines OOP payments were estimated to be 6.77% [CI: 6.70%, 6.84%] and 4.49% [4.45%, 4.54%] of total consumption expenditure respectively in the year 2011-12 which marked significant increase since 1993-94. These proportions were 11.46% [CI: 11.36%, 11.56%] and 7.60% [CI: 7.54%, 7.67%] of non-food expenditure respectively in the same year. It was observed that total OOP payments and medicines OOP payments were catastrophic for 17.9% [CI: 17.7%, 18.2%] and 11.2% [11.0%, 11.4%] households respectively in 2011-12 at the 10% of total consumption expenditure threshold, implying 29 million households incurred catastrophic out-of-pocket payments in the year 2011-12. Further, medicines out-of-pocket payments pushed 3.09% [CI: 2.99%, 3.20%], implying 38 million, persons into poverty in the year 2011-12. Among the leading cause of diseases that caused significant OOP payments are cancers, injuries, cardiovascular diseases, genito-urinary conditions and mental disorders.

Conclusions: Purchase of medicines constitute the single largest component of the total OOP payments by households. Hence, strengthening government intervention in providing medicines free in public health care facilities has the potential not only to considerably reduce medicines related spending and total OOP payments of households but also reduction in OOP induced poverty.

Key words: India, out of payment, medicine, catastrophic expenditure, and poverty

For peer review only

Article summary

Strengths and limitations of this study

1. The study used multiple points of time and nationally representative data set to highlight the financial burden of households OOP payments on medicines in India;
2. The paper links medicines out-of-pocket payments by households with leading disease conditions and identify key disease conditions which cause medicines out-of-pocket payments
3. The study has limitations as it uses arbitrary threshold for measuring catastrophic payments
4. The ailments, disease conditions and the associated OOP expenditure reported by the households in the survey are self-reports and not clinically diagnosed.

For peer review only

Background

Households' in India bear significant financial burden on account of medical treatment, as the current prepayment and risk pooling mechanisms are inadequate. Since both government funding and social health insurance contributions are insufficient to meet health care needs of households, over three-fourth of all health care payments are paid out-of-pocket (OOP) at the point of service delivery while medicines purchase (approximately 63%) account for the single largest component of these payments.[1] Available literature suggests that OOP spending has dominated total OOP payments over the years.[2] Hence, it can be suggested that expenditure on medicines is major cause of catastrophe at the household level.[3]

India has the distinction of being pharmacy of the global south - supplies affordable, life saving, quality generic medicines. It ranks 4th in terms of volumes and 13th in terms of value of pharmaceuticals produced globally.[4] However, according to a WHO report around 68% of the Indian population have limited or no access to essential medicines.[5] In addition, literature suggests that over last two decades availability of free medicines in public health facilities has declined from 31.2% to 8.9 % for inpatient care and from 17.8% to 5.9% for outpatient care.[6] Another study demonstrated that medicines purchase alone constituted over 70% of overall OOP payments. In addition, the study demonstrated that by removing OOP payments for outpatient care on medicines, the percentage of people falling below poverty because of spending on health reduced to just 0.5 % whereas removing OOP payments for inpatient care resulted in a negligible decline in poverty headcount ratio and poverty gap highlighting the role of medicines expenditure in healthcare related impoverishment.[7]

Utilising impoverishment tool to measure affordability, one study assessed the impoverishment effect of medicines purchases by households in 16 low-and-middle income economies.[8] Comparing four key medicine prices to household income, and using World Bank poverty levels of US$ 1.25 or US$ 2 per day, the study concluded that a substantial number of people had to bear financial burden due to unaffordability of medicines. For example, it was pointed out that an originator brand atenolol purchase by individuals would push an additional 22% of population below the US$ 1.25 per day measurement while even a generic equivalent of atenolol was likely to push about 7% of population below poverty levels in Philippines.[9] Analyzing economic implications of non-communicable disease in India, a few studies also reported in the past that households incur significant OOP payment burden in certain conditions like cardiovascular diseases (CVD) and cancers.[10 11] Using 2004 NSSO data, another study highlighted that hospitalization with CVD resulted in 12% higher odds of incurring catastrophic spending and 37% greater odds of falling into poverty compared to those hospitalized with communicable conditions. For cancer, the impact was greatest with the odds of catastrophic expenditures 170% higher than the odds of incurring catastrophic spending when hospital stays are due to a communicable disease condition.[12]

For peer review only - http://bmjopen.bmj.com/site/about/guidelines.xhtml

However these studies do not reflect on the relative contribution of medicine in total OOP burden for the diseases they analysed.

Available evidence, both global and Indian, provides insights about the incidence of catastrophic payments and impoverishment impact of rising households OOP payments.[2 3 13-15] The literature on equity dimensions involving both catastrophe and impoverishment has attempted to address complex methodological and statistical approaches in measurements. However, there is lack of evidence on catastrophe and impoverishment on account of household's medicine OOP expenditure not only from inpatient and outpatient treatment costs perspective but also from the disease specific dimension. Given that medicines contribute to more than 66.6% of out-of-pocket healthcare expenditure, the focus of this research is to explore the consequences of high medicines OOP spending at the household level. Further, we investigated which disease conditions are contributing to high financial burden on households. We attempted to answer - what is the relative burden of medicines OOP payments by households in total OOP payments, catastrophic and poverty headcounts? And which disease conditions cause a relatively higher financial disruption in the living status of households?

Materials and methods

Data

The study utilises secondary data from three waves of nationally representative 'Consumer Expenditure Surveys' (CES): 1993-94, 2004-05 and 2011-12, conducted by the National Sample Survey Organisation (NSSO). In addition, health and morbidity survey (HMS) 2014 of the NSSO was used for disease-wise distribution of OOP payment burden. While the sample size of CES varied between 100,000 to 125,000 thousand households across different rounds, the sample size in HMS 2014 was approximately 72,000 households.

The CES collect socioeconomic and demographic information on households with key focus is on household spending on roughly 350 food and non-food items. Out-of-pocket medical expenses incurred by households are separately recorded for inpatient and outpatient services. The recall periods are one-year and 30-days for inpatient and outpatient expenses respectively. HMS collects detailed information on morbidity pattern, utilisation of health care services and associated expenditure by households. The HMS too separately records expenditure for inpatient and outpatient. However, unlike in CES the recall period for outpatient in HMS is 15 days.

Both CES and HMS are repeated cross-section surveys that are representative at the national and state levels. All districts of a state are included for sampling purposes. Households in CES are sampled evenly in quarterly sub-rounds beginning on 1 July and ending on 30 June of the following year, with equal numbers of households allotted in each quarterly sub-round, to address seasonality. In HMS, survey was completed in two sub-

rounds during January1 to June 30, 2014. All estimates in the present paper are sample weighted.

Outcome indicators

Using CES, we estimated 4 household-level indicators involving financial burden of illness: i) per household member monthly OOP spending on medicines (inflation-adjusted), ii) OOP spending on medicines as a share of total household and non-food spending, and iii) percentage of households reporting catastrophic payments on medicines and iv) percentage of households slipping onto poverty after netting out medicines OOP payments from households' total consumption expenditure. Total OOP spending of households was estimated by adding together expenditure on components of OOP payments. For inpatient OOP payment, we considered institutional spending on medicines, X-ray, ECG, pathological tests etc., doctor's/surgeon's fee, hospital and nursing charges and other medical expenses. For outpatient OOP payment, the components of expenditure are medicines, X-ray, ECG, pathological tests etc., doctor's/surgeon's fee, family planning services and other medical expenses. Expenditure on medicines is directly reported in the data set, both for inpatient and outpatient services (see Table A-I in Annexure). All the analyses report mean OOP spending on two parallel tracks: aggregated (across components of OOP) OOP payments (henceforth referred to as 'total OOP') and OOP payments only on account of medicine purchase (henceforth referred to as medicine/drug OOP).

Catastrophic payment for health care is defined as OOP payments being higher to a pre-defined threshold of total household consumption expenditure or alternatively household's non-food expenditure. For measuring catastrophic expenditure,[2 9] instead of sticking to a particular threshold, we considered a range of thresholds.[2 16-18] We also considered alternative thresholds as OOP spending as a share of household non-food expenditure. For OOP payment induced poverty estimates, we used two different poverty lines: i) Indian official state-specific rural and urban poverty lines[19] and ii) international poverty line based on US $ 1 per day per person adjusted to US $ 1.9 purchasing power parity (PPP) per day per person for the year 2011-12[20]. Yet another important poverty indicator, which particularly estimates magnitude of poverty deepening, is poverty gap. Using both the poverty lines separately, we also estimated mean poverty gaps for the poor. Details of the method used for catastrophic and poverty estimates are presented in Annexure.

In addition, we used NSSO 2014 HMS data for estimating disease level total and medicine OOP spending separately for inpatient and outpatient. Unlike CES, OOP spending in HMS 2014 has not been recorded as part of the total household consumption expenditure and instead of estimating disease-wise catastrophic headcount, we present distribution of disease conditions based on incidence of occurrence and range of OOP spending separately for outpatient (15 days recall converted for 30 days) and inpatient (365 days recall

For peer review only - http://bmjopen.bmj.com/site/about/guidelines.xhtml

converted for 30 days). This helped identifying disease conditions, separately for outpatient and inpatient, which are high frequency occurrence and greater incidence of OOP spending.

Results

First, we present basic financial burden indicators for the years 1993-94, 2004-05 and 2011-12 in Table 1. Over 80% of populations are reportedly spending out-of-pocket while seeking treatment, during 2011-12. The proportion of population reporting any OOP payments have increased sharply from about 60% during 1993-94 to 80% percent in 2011-12. In respect to medicines spending, approximately every OOP spending is associated with expenditure on medicines. There was a significant increase (more than 50%) in household's total consumption expenditure in real terms from INR 517 in 1993-84 to INR 794 in 2011-12. However, during the same period total OOP payments increased by more than 100% from INR 26 in 1993-94 to INR 54 in 2011-12 in real terms. The rise in OOP payments on medicines has been more than 70% during the same period. Consequently, the share of spending on health from households' overall consumption expenditure have registered sharp increase during the past two decades, from a moderate 4.8% during 1993-94 to nearly 7% in 2011-12. If we were to net out food expenditure from total household consumption spending, which are considered a necessity, the share of health spending remained stagnant but as high as 11-12% percent during the period under consideration. It may be observed that in 2011-12 medicines alone contributed up to 67% of the total OOP payments.

Table 1: **Financial burden indicators, India, 1993-94, 2004-05 and 2011-12**

**Financial burden indicators**	**1993-94**	**2004-05**	**2011-12**
**Percentage households reporting OOP payments**			
Any out-of-pocket payments (%)	59.2 [58.9, 59.5]	64.4 [64.2, 64.7]	80.5 [80.2, 80.7]
Medicines out-of-pocket payments (%)	57.5 [57.3, 57.8]	63.6 [63.3, 63.8]	79.0 [78.8, 79.3]
**Monthly per capita expenditure (INR at constant 1999-2000 prices*)**			
Household consumption expenditure	517 [515, 519]	619 [616, 622]	794 [790, 799]
Out-of-pocket expenditure on health	25.59 [24.61, 26.25]	36.3 [35.7, 37.0]	54.3 [53.3, 55.3]
Medicine out-of-pocket expenditure	20.86 [19.50, 21.25]	26.0 [25.6, 26.4]	36.1 [35.5, 36.8]
**Share of health to total household expenditure (%)**			
Share of total out-of-pocket expenditure to total household expenditure (%)	4.84 [4.78, 4.91]	5.78 [5.72, 5.83]	6.77 [6.70, 6.84]

For peer review only - http://bmjopen.bmj.com/site/about/guidelines.xhtml

Share of medicine out-of-pocket expenditure to total household expenditure (%)	3.93 [3.87, 3.98]	4.10 [4.06, 4.14]	4.49 [4.45, 4.54]
**Share of health to non-food household expenditure (%)**			
Share of total OOP payments to non-food expenditure (%)	12.37 [12.20, 12.55]	10.82 [10.72, 10.91]	11.46 [11.36, 11.56]
Share of medicines OOP payments to non-food expenditure (%)	10.02 [9.88, 10.17]	7.68 [7.62, 7.75]	7.60 [7.54, 7.67]

Notes: 1. numbers in brackets are 95% confidence interval; 2. State and rural-urban specific consumer price indices were used to convert current prices values at the constant 1999-2000 prices. 3. The current prices values for monthly per capita total OOP are 15.7, 41.8 and 111.2 and for medicine OOP are 12.8, 29.8 and 73.9 (all in INR) for the years 1993-94, 2004-05 and 2011-12 respectively.

A higher burden of households' OOP payment is often associated with impoverishment and catastrophe. In Table 2, we present a set of catastrophic cut-offs measured as a share of OOP payments to total household consumption expenditure and non-food expenditure. Estimates for both total OOP payments as well as medicine OOP payments by households are presented.

Table 2: Percentage of households incurring catastrophic payments with respect to total OOP spending and medicines OOP spending, 1993-94, 2004-05 and 2011-12

**Financial Health Equity Measurements**	1993-94 (%)	2004-05 (%)	2011-12 (%)	Estimated number of households (2011-12)
**Cut-off for catastrophe using total household expenditure**				
Total OOP payment > 5%	26.9 [26.6, 27.1]	28.7 [28.5, 30.0]	35.3 [35.0, 35.6]	9,01,07,225
Total OOP payment > 10%	13.9 [13.8, 14.2]	14.6 [14.4, 14.8]	17.9 [17.7, 18.2]	4,56,91,766
Total OOP payment > 25%	3.9 [3.8, 4.0]	3.5 [3.4, 3.6]	4.3 [4.2, 4.4]	1,09,76,234
Medicines OOP payment > 5%	23.3 [23.0, 23.5]	23.4 [23.2, 23.6]	27.0 [26.7, 27.2]	6,89,20,540
Medicines OOP payment > 10%	11.5 [11.3, 11.7]	10.2 [10.2, 10.4]	11.2 [11.0, 11.4]	2,85,89,261

For peer review only - http://bmjopen.bmj.com/site/about/guidelines.xhtml

Medicines OOP payment > 25%	02.9 [2.8, 2.9]	1.6 [1.5, 1.7]	1.8 [1.7, 1.9]	45,94,703
**Cut-off for catastrophe using non-food expenditure**				
Total OOP payment > 5%	47.8 [47.5, 48.1]	46.5 46.2, 46.8]	53.5 [53.2, 53.8]	13,65,64,775
Total OOP payment > 10%	34.8 [34.6, 35.1]	31.0 [30.7, 31.2]	34.9 [34.7, 35.2]	8,90,86,180
Total OOP payment > 25%	16.7 [16.5, 16.9]	11.4 [11.2, 11.5]	11.9 [11.7, 12.1]	3,03,76,090
Total OOP payment > 40%	9.7 [9.5, 9.9]	4.7 [4.6, 4.9]	4.9 [4.8, 5.0]	1,25,07,802
Medicines OOP payment > 5%	44.7 [44.4, 45.0]	42.5 [42.2, 42.8]	46.4 [46.1, 46.7]	11,84,41,225
Medicines OOP payment > 10%	31.2 [31.0, 31.5]	25.5 [25.3, 25.7]	26.1 25.9, 26.4]	6,66,23,189
Medicines OOP payment > 25%	13.9 [13.7, 14.1]	7.1 [7.0, 7.3]	6.3 [6.1, 6.4]	1,60,81,459
Medicines OOP payment > 40%	7.8 [7.6, 7.9]	2.2 [2.1, 2.3]	1.8 [1.7, 1.9]	45,94,703

Note: Figures in brackets are 95% confidence interval.

For peer review only

Over one-third of Indian households incurred OOP payments greater to 5% of total household expenditure in 2011-12. This percentage was lower in 1993-94 (27%) and 2004-05 (28%). At the 25% threshold of total household expenditure, over 4% households reported incurring OOP payments in 2011-12. This essentially translates to approximately 11 million Indian households. Out of these, more than 4.4 million households incurred such payments only on account of purchase of medicines. At a lower threshold of 10% of total household expenditure, the number of households facing catastrophe is approximately 46 millions, of which 29 million households incurred catastrophe on account of OOP payments on medicines alone. Considering only non-food expenditure of households as the basic living status variable, approximately similar number of households incurred medicines OOP payments in 2011-12 with OOP payments being as high as 40% of their non-food expenditure.

For peer review only - http://bmjopen.bmj.com/site/about/guidelines.xhtml

Table 3: Impoverishment Indicators due to households' total OOP and medicine spending, India, 1993-94, 2004-05 and 2011-12

	1993-94	2004-05	2011-12	Estimated population in millions, (2011-12)
**I. Using national poverty line***				
1. Headcount ratio indicators (%)				
Gross Headcount	45.32 [45.03, 45.61]	37.85 [37.58, 38.12]	22.17 [21.92, 22.43]	272
Headcount net of total OOP	49.52 [49.22, 49.81]	42.68 [42.40, 42.95]	26.65 [26.38, 26.92]	327
Total OOP payment induced poverty	4.20 [4.07, 4.30]	4.83 [4.71, 4.94]	4.48 [4.35, 4.60]	55
Headcount net of medicine OOP payment	48.91 [48.61, 49.20]	41.54 [41.27, 41.82]	25.27 [25.00, 25.53]	310
Medicine OOP payment induced poverty	3.59 [3.47, 3.69]	3.69 [3.59, 3.80]	3.09 [2.99, 3.20]	38
2. Poverty gap indicators (INR current prices)				
Gross poverty gap#	63.3 [62.9, 63.8]	103.4 [102.7, 104.2]	154.2 [152.3, 156.0]	
Gap net of total OOP payment ##	69.7 [69.3, 70.1]	115.8 [115.1, 116.5]	182.8 [181.0, 184.7]	
Total OOP payment induced gap##	6.4 [6.3, 6.5]	12.4 [12.2, 12.6]	28.6 [28.0, 29.2]	
Gap net of medicine OOP payment ###	68.9 [68.5, 69.3]	113.7 [113.0, 114.4]	176.7 [174.9, 178.5]	
Medicine OOP payment induced	5.6 [5.5, 5.7]	10.3 [10.1, 10.4]	22.5 [22.0, 23.0]	

For peer review only - http://bmjopen.bmj.com/site/about/guidelines.xhtml

gap###				
**II. Using international poverty line****				
1. Headcount ratio indicators (%)				
Gross Headcount	40.96 [40.67, 41.24]	33.07 [32.81, 33.34]	18.37 [18.13, 18.61]	225
Headcount net of total OOP payment	44.92 [44.63, 45.21]	37.38 [37.11, 37.65]	22.41 [22.16, 22.67]	275
Total OOP payment induced poverty	3.97 [3.85, 4.08]	4.31 [4.19, 4.42]	4.04 [3.92, 4.16]	50
Headcount net of medicine OOP payment	44.35 [44.06, 44.64]	36.34 [36.08, 36.61]	21.37 [21.11, 21.62]	262
Medicine OOP payment induced poverty	3.39 [3.29, 3.50]	3.27 [3.17, 3.68]	2.99 [2.89, 3.10]	37
2. Poverty gap indicators (INR current prices)				
Gross poverty gap#	59.3 [58.9, 59.7]	96.1 [95.3, 96.8]	150.7 [148.8, 152.7]	
Gap net of total OOP payment ##	65.4 [64.9, 65.8]	107.5 [106.8, 108.3]	177.0 [175.1, 179.1]	
Total OOP payment induced gap##	6.1 [6.0, 6.2)	11.5 [11.2, 11.7]	26.3 [25.7, 27.0]	
Gap net of medicine OOP payment ###	64.6 [64.2, 65.1]	105.8 [105.0, 106.5]	172.0 [170.0, 174.0]	
Medicine OOP payment induced gap###	5.3 [5.2, 5.4]	9.7 [9.5, 9.9]	21.3 [20.7, 21.8]	

Notes: based on Tendulkar Committee methods (poverty lines range between INR 695 in Odisha and INR 1018 in Kerala in rural and INR 861 in Odisha and INR 1169 in Haryana in urban areas among the major states); ** using USD 1.90 PPP at 2011-12 prices and mixed recall period of household consumption expenditure (INR equivalent to USD 1.90 PPP are 771.21 in rural and 945.41 in urban areas); # only for poor; ## only for poor net of total OOP; ### only for poor net of medicine OOP

For peer review only - http://bmjopen.bmj.com/site/about/guidelines.xhtml

Next, we present implications of total and medicine OOP payments on poverty estimates (Table 3). To facilitate interpretation, we present 3 basic headcount ratio indicators: i) gross headcount – percentage of population below poverty line, ii) net of OOP headcount – percentage of population below poverty line after netting out OOP payments from household consumption expenditure and iii) OOP payments induced poverty which is the difference of the first two reflecting rise in poverty ratio owing to OOP payments. The last two indicators are presented separately for total OOP payments and medicine OOP payments. Table 3 also provides estimates on poverty gap representing extent of poverty deepening in terms of monetary value. All these indicators are estimated using Indian official poverty line (Tendulkar Committee method) and international poverty line of US $ 1.90 PPP.

The difference in mean headcount measure of gross and net poverty ratios reflects the percentage of population falling below poverty line because of households' OOP payments on health care. The headcount ratio of households impoverished due to OOP payments was 3.97% during 1993-94, which inched up to 4.30% in 2004-05 while in 2011-12 it was at 4.04%, as per international poverty line. In terms of Indian state-specific official poverty lines, percentage of households falling below poverty line increased from 4.19% in 1993-94 to 4.48% in 2011-12. This translates to 55 million persons in 2011-12. Out of this, approximately 38 million became poor only because they had to purchase medicines through OOP payments. Using the same measurement, the headcount measure for households OOP payments on medicines appear to have marginally declined from 3.58% in 1993-94 to 3.09% during 2011-12 using the international poverty line. As far as poverty gap is concerned, based on the Indian official poverty line, total OOP payments and OOP payments on medicines resulted in poverty deepening among poor by INR 29 and INR 23 respectively in 2011-12. Further poverty deepening because of total and medicines OOP payments sharply increased in 2012 compared to that in the years 2004-05 and 1993-94.

OOP expenditure by disease conditions

We also conducted a disaggregated analysis on disease wise expenditure not only with reference to total OOP payments and medicines OOP payments but also by type of care - inpatient care versus outpatient care. The survey results suggested that most common health condition for seeking outpatient care was fever (22.7%) and for inpatient care was childbirth (27.3%). In addition, our estimates suggest that households incurred highest monthly per capita OOP spending both for inpatient and outpatient care on account of cancer treatment (INR 5,054 and INR 5,121 respectively) followed by injuries for outpatient care (INR 3,045) and cardiovascular events for inpatient care (INR 2,808).

We also mapped disease-wise expenditure, frequency of healthcare utilization and type of care to demonstrate that not only hospitalization but also outpatient care can lead to catastrophe and impoverishment of households (Figure 1). For example, our estimates

For peer review only - http://bmjopen.bmj.com/site/about/guidelines.xhtml

suggest that monthly per capita medicines OOP payments for cancer care were significantly higher in outpatient care as compared to the inpatient care. However, as far as total OOP spending for cancer treatment is concerned, it is almost similar across inpatient and outpatient but significantly higher compared to that for other disease conditions. In contrast, in respect to cardiovascular conditions, medicines OOP payments were similar for both inpatient and outpatient treatment but total OOP payments were significantly higher for inpatient treatment as against outpatient treatment. In treatments involving gastroenterology conditions, however, both medicines OOP payments and total OOP payments were higher for outpatient compared to inpatient treatment. Similarly, for mental disorders, medicines OOP payments were higher for outpatient care compared to inpatient but total OOP payments were almost similar both for outpatient and inpatient treatment. Therefore, it is noted that the average monthly medicines OOP payments were consistently higher for outpatient care as compared to inpatient care among key disease conditions. A relatively higher frequency of outpatient treatment visits compared to inpatient treatment coupled with a significantly larger medicines OOP payment may yield a higher incidence of catastrophe. A detailed estimate of prevalence and OOP payments by disease conditions cross-classified by inpatient and outpatient care are presented in Table A-II in Annexure.

Further, we plotted outpatient and inpatient OOP payments and medicines related OOP payments with respect to households 'usual' consumption expenditure. In Figure 2, households are ranked from the poorest to the richest on the X-axis based on their mean monthly per person consumption expenditure and on the Y-axis mean monthly per person OOP expenditure (total and medicine) are measured separately for outpatient and inpatient. It is observed that for a number of households, average monthly outpatient expenditure is not only significantly higher in relation to household's non-medical consumption expenditure but the frequency of such events is also higher in outpatient care as compared to inpatient care. In Figure 2, the concentration of red (total OOP payment) and green (medicine OOP payment) spikes above the consumption expenditure on the right hand side of the graph which reflects that even among richer households total OOP and medicine OOP payments are significantly higher than total non-medical consumption expenditure of households. Moreover, concentration of medicine OOP payments above households' non-medical consumption expenditure is more prominent in case of outpatient compare to the inpatient episodes.

Discussion and conclusion

Using standard methods of measuring catastrophe and impoverishment, [17 18] this paper demonstrates the financial burden of households' OOP payments on medicines in India, spanning two decades from 1993-94 to 2011-12. To our knowledge, this is a first attempt to link medicines' OOP spending to key diseases conditions. Two trends stand out clearly from our findings. First, the households' impoverishment on account of OOP expenditure is

For peer review only - http://bmjopen.bmj.com/site/about/guidelines.xhtml

rather high and continued to be so during the last two decades. The impoverishment burden is largely driven by households spending on medicines, which accounted for over three-fourth of all medical impoverishment in India. One of the reasons could be compared to OOP payments on medicines, hospitalization/bed charges in India are comparatively low, and often subsidized in the public sector. Second, as far the catastrophe measurement is concerned, applying a 10% threshold of OOP payment on overall consumption expenditure, an estimated 18% percent of Indian households appear to suffer financial catastrophe. Medicines' OOP expenditure alone contributed to an estimated 11% of financial catastrophe. In absolute numbers, this translates to a scenario where an estimated 46 million households appear to face catastrophic expenditure on account of OOP payments while 29 million households faced such hardship because they had to pay for medicines from their pockets.

Recent evidence from the National Health Accounts for India points out that during 2013-14, an estimated INR 1,331 per capita was spent on medicines, while households alone contributed INR 1,200 per capita, accounting for 90% of all medicines expenditure in the country.[1] On the other hand, past evidence about government expenditure on medicines in India, underscores that, on an average government spent about 10% percent of health expenditure on medicines. However, the national average masks significant underspending on medicines by several state governments, with many reportedly spending less than five percent of their health budgets.[6] Besides poor allocation of resources, except for a couple of Indian states, drug procurement and supply chain system is inefficient and ineffective leading to acute shortages of key essential medicines and chronic stock-outs in public health facilities.[21-24] This situation has resulted in physical unavailability of medicines. Drawing evidence from large sample surveys for the period from 1986-87 to 2004, it is reported the physical barrier to access to key essential medicines worsened during this period.[15] Supply of free drugs in government health system in the outpatient care setting, declined sharply from about 18% in 1986-87 to 5% in 2004. For the same period, drugs prescribed during hospitalization for free also declined significantly from one-third to about 9%.[6] As a result, it is pointed out the number of hospitalization episodes in which an ailing population paid out-of-pocket (OOP) payment, has risen dramatically from about 41% to close to 72%.[6] Further, it was observed that from the period spanning mid-1990s to 2004, patients visiting government health facilities did not receive medicines in over one-fourth of outpatient episodes. Affordability of medicines is an important access indicator, because it translates into poor access or no access for people who have low purchasing power.[3] The consumer behaviour theory also predicts that raising the price (via high OOP expenditure on medicines or high copayment) for a service in the public health sector will move more consumers into the private sector, depending on the elasticity of substitution and transaction costs in the public sector.[25]

For peer review only - http://bmjopen.bmj.com/site/about/guidelines.xhtml

In view of inadequate availability of medicines in government health facilities, households end up accessing private facilities where they end up incurring significant OOP payment, in the absence of any financial risk protection. Past evidence suggests that the trend has sharpened in the last couple of decades. For instance, the percentage of population accessing private facilities for inpatient and outpatient treatment has accelerated significantly between 1986-87 and 2004. It may be observed that households accessing private hospital for inpatient care increased from around 40% to nearly 60% in rural India while urban India reported a rise from 40% to 68%.[26] During the same period, outpatient care visits in private facilities remained high at around 75% in 1986-87 in rural India and 73% in urban India stepped up to 78% and 80% respectively for rural and urban India.[3]

The other critical evidence emerging from this paper focuses on disease specific medicines expenditure. The results demonstrate a pattern where households' medicine spending is concentrated on low frequency, high-value spending and high frequency, high-value spending. By disease-wise classification, expenditure on treatment of cancers, CVDs and injuries, both for outpatient and inpatient care dominate the spending pattern. Available literature confirms such an expenditure pattern, wherein the share of non-communicable diseases (cardiovascular disease, diabetes, cancer, mental illness, injuries and others) in OOP health expenses has increased from 31.6% in 1995-96 to 47.3% in 2004.[12] The literature further indicates high odds of catastrophic hospitalization expenditures for certain NCDs. For example, the odds for catastrophic expenditure in cancer are nearly 170% greater, for cardiovascular diseases (CVD) and injuries nearly 22% greater than the odds due to infectious diseases. Other studies on cardiovascular diseases highlighted that CVD affected households had more outpatient visits and inpatient stays, spent extra money per hospitalization [11] and have high probability of incurring catastrophic expenditure compared to those using inpatient facilities for communicable conditions.[27] Another Indian study on socio-economic inequalities in financing of diabetes and cardiovascular disease reported that out-of-pocket payments for hospital treatment claimed a large share of annual household expenditures; 30% for CVD and 17% for diabetes.[28] In respect to injuries (both road traffic and non-road traffic), high incidence of catastrophic expenditure was 30%, and was significantly higher among those belonging to the lowest income quartile and with an inpatient stay greater than 7 days.[29]

Although public facilities have slightly stepped up their share in outpatient care in recent years, private sector continue to dominate both in outpatient and inpatient care in India.[30] As an increasing share of households' access private health facilities, private retail pharmacies have become a major source of supply of key essential medicines. While availability of medicines may per se is not a challenge in the private health care setting, affordability appears to act as critical barrier.[31] Thus, pricing of medicines and regulation around retail medicine prices becomes a key factor in improving affordability and thereby leading to a reduction in medicines related OOP payment burden. Although India had a

For peer review only

progressive retail price cap policies since 1979, but over the years a policy of deregulation was followed.[32] In 2013, the Government of India promulgated the Drugs Price Control Order (DPCO), 2013 (DPCO, 2013) which primarily brought all essential drugs, based on National List of Essential Medicines, 2011, under price control.[33] An evaluation of new price regulation has highlighted that, while few of the medicines (37) had an increase in sales volume attributable to DPCO, majority of the medicines (52) had a negative impact on their sales volume due to DPCO. Overall, the DPCO may have had a negative impact in terms of sales volume of medicines under price control.[34] Given that the sales volume of price-capped medicines has declined, households OOP spending may continue to increase since over 80% of retail pharmacy market is not price-capped.

In order to improve access to health care and to provide financial risk protection to households, the central government and several state governments have been implementing a publicly funded health insurance programs since 2007, whose primary aim was to provide cashless treatment to economically vulnerable households for hospitalization episodes. Emerging evidence from micro as well as macro level studies point to a trend where such insurance schemes appear to have improved access to hospital care but have been ineffective in preventing financial catastrophe and impoverishment to households.[2 35 36] These bodies of evidence are in line with our findings that hospitalization based treatment cost constitute only one-third of India’s morbidity burden. Despite implementation of several health insurance schemes, a majority of Indian population continues to incur a relatively significant medicines OOP payment while seeking outpatient care. It would be pertinent to highlight that the frequency of hospitalization is considerably smaller than outpatient visits in general, especially for non-communicable diseases (NCDs), which are chronic in nature that require multiple consultations and long-term or life-long medication support. Such medical conditions result in catastrophic expenditure for households even in the absence of hospitalization episodes. Moreover, since a relatively larger proportion of population seeks outpatient care in private facilities, which is often multiple times expensive than public health facilities, we observe a disproportionately higher burden of medicines related OOP payment for outpatient care.

The evidence presented in this paper, however, suffers from a few limitations. The first set of challenge relates to co-morbidities and associated expenditure. In respect to inpatient cases, since NSSO data captures disease expenditure separately for various disease conditions, the issue of co-morbid conditions did not play major role. However, for outpatient cases, we had to adopt apportioning technique to handle co-morbid conditions. The second set of challenge pertains to the potential recall bias for disease specific expenditures, which cannot be ruled out especially for hospitalization treatment since the recall period is a longer time span of 365 days. Lastly, although there are significant state level and rural-urban differentials in the estimates presented in this paper, we focused on

For peer review only

the All India average and believe that the state level and rural-urban analyses could be a potential research for the future.

The foregoing underlines several policy interventions and program design that were conceived and implemented in the recent past to provide financial risk protection to households. However, gross underinvestment in the public health system in past had led to inadequate prepayment and risk pooling measures.[26] Several policy interventions and program redesign are required to reverse the trend of high OOP expenditure for healthcare in India. An efficient and a reliable medicines supply chain model existed for over two and half decades in the state of Tamil Nadu, which was replicated in the state of Rajasthan in 2012 have been instrumental in improving access to medicines in the frontline facilities in these two states.[37] Such policies and programs governing public health facilities are critical. The National Health Policy 2017 also highlighted the need for providing free medicines in public health facilities by stepping up funding and improving drug procurement and supply chain mechanisms.[38] A recent pronouncement by the government intends to bring legislation for physicians to prescribe drugs only in generic names, also holds promise for reducing households' OOP payments on medicines and ultimately providing financial risk protection. To sum up, both national and state governments' intervention is required for providing free medicines in public health facilities along with expanding the mechanism of price capping of key essential medicines in the private market.

Contributorship: AK and SS conceived the idea, designed the analysis, conducted data analysis and wrote the first draft of the paper. AK, SS and HHF conducted the literature review and the interpretation of the results. AS, SS and HHF revised and edited the manuscript to its final stages. All the authors approved the final manuscript version.

Acknowledgments: None

Competing Interests: We declare no conflict of interest.

Funding: Habib Hasan Farooqui is supported by a Welcome Trust Capacity Strengthening Strategic Award to the Public Health Foundation of India and a consortium of UK universities. The funders had no role in study design, data collection and analysis, decision to publish, or preparation of the manuscript.

Data sharing: The data used for the analysis is available in public domain.

Ethical approval: Ethical approval for this study was not needed. The study used only anonymised data from secondary sources. Requisite permission to use the data has been obtained from the agency.

For peer review only

Reference list:

1. (NHSRC). NHSRC. National Health Accounts Estimates for India 2013-14: Ministry of Health and Family Welfare (MoHFW). Government of India, 2014.
2. Karan A, Selvaraj S, Mahal A. Moving to universal coverage? Trends in the burden of out-of-pocket payments for health care across social groups in India, 1999-2000 to 2011-12. *PloS one* 2014;9(8):e105162. doi: 10.1371/journal.pone.0105162
3. Selvaraj S, Karan A. Deepening health insecurity in India: evidence from national sample surveys since 1980s. . *Econ Polit Wkly* 2009;44:55–60.
4. WHO. The World Medicines Situation 2011. Geneva: World Health Organozation, 2011.
5. WHO. The World Medicine Situation 2004. Geneva: World Health Organization, 2004.
6. Reddy K. High Level Expert Group (HLEG) report on Universal Health Coverage (UHC) for India. New Delhi: Public Health Foundation of India, 2011.
7. Shahrawat R, Rao KD. Insured yet vulnerable: out-of-pocket payments and India's poor. *Health policy and planning* 2012;27(3):213-21. doi: 10.1093/heapol/czr029
8. Khatib R, McKee M, Shannon H, et al. Availability and affordability of cardiovascular disease medicines and their effect on use in high-income, middle-income, and low-income countries: an analysis of the PURE study data. *Lancet* 2016;387(10013):61-9. doi: 10.1016/S0140-6736(15)00469-9
9. van Mourik MS, Cameron A, Ewen M, et al. Availability, price and affordability of cardiovascular medicines: a comparison across 36 countries using WHO/HAI data. *BMC Cardiovasc Disord* 2010;10:25. doi: 10.1186/1471-2261-10-25
10. Mahal A, Karan A, Fan VY, et al. The economic burden of cancers on Indian households. *PloS one* 2013;8(8):e71853. doi: 10.1371/journal.pone.0071853
11. Karan A, Engelgau M, Mahal A. The household-level economic burden of heart disease in India. *Trop Med Int Health* 2014;19(5):581-91. doi: 10.1111/tmi.12281
12. Engelgau MM, Karan A, Mahal A. The Economic impact of Non-communicable Diseases on households in India. *Globalization and health* 2012;8:9. doi: 10.1186/1744-8603-8-9
13. Khan JAM, Ahmed S, Evans TG. Catastrophic healthcare expenditure and poverty related to out-of-pocket payments for healthcare in Bangladesh-an estimation of financial risk protection of universal health coverage. *Health policy and planning* 2017;32(8):1102-10. doi: 10.1093/heapol/czx048
14. Ghosh S. Catastrophic Payments and Impoverishment due to Out-of-Pocket Health Spending. *Econ Political Wkly* 2011;46(47)
15. Selvaraj S, Karan A. Deepening health insecurity in India: evidence from national sample surveys since 1980s. . *Econ Political Wkly* 2009;44:55–60.
16. Fan VY, Karan A, Mahal A. State health insurance and out-of-pocket health expenditures in Andhra Pradesh, India. *Int J Health Care Finance Econ* 2012;12(3):189-215. doi: 10.1007/s10754-012-9110-5
17. Van Doorslaer E ODO, Rannan-Eliya RP, Somanathan A, Adhikari SR, Garg CC, et al. . Effect of payments for health care on poverty estimates in 11 countries in Asia: an analysis of household survey data. *The Lancet* 2006;368:1357-64.
18. Xu K, Evans, D.B., Kawabata, K., Zeramdini, R., Klavus, J., Murray, C.J.L. Household catastrophic health expenditure: a multicountry analysis. *Lancet* 2003;362 111–17.
19. GOI. Report of the Expert Group to Review the Methodology for Measurement of Poverty. New Delhi.: Planning Commission., 2014.

For peer review only - http://bmjopen.bmj.com/site/about/guidelines.xhtml

20. Bank W. World Bank 2017 [Available from: http://iresearch.worldbank.org/PovcalNet/FAQs.aspx; 2017.
21. Selvaraj S, Chokshi, M., Hasan, H., & Kumar, P. . Improving Governance and Accountability in India's Medicine Supply System. . New Delhi: Public Health Foundation of India., 2010.
22. Prinja S, Bahuguna P, Tripathy JP, et al. Availability of medicines in public sector health facilities of two North Indian States. *BMC Pharmacol Toxicol* 2015;16:43. doi: 10.1186/s40360-015-0043-8
23. Kotwani A, Gurbani N, Sharma S, et al. Insights for policymakers from a medicine price survey in Rajasthan. *Indian J Med Res* 2009;129(4):451-4.
24. Kotwani A. Where are we now: assessing the price, availability and affordability of essential medicines in Delhi as India plans free medicine for all. *BMC health services research* 2013;13:285. doi: 10.1186/1472-6963-13-285
25. Alsan M SL, Eggleston K, Kammili N, Kolli P, Bhattacharya J. . Out-of-pocket health expenditures and antimicrobial resistance in low-income and middle-income countries: an economic analysis. . *Lancet Infect Dis* 2015;15(10):(10):1203-10.
26. Mackintosh M, Channon A, Karan A, et al. What is the private sector? Understanding private provision in the health systems of low-income and middle-income countries. *Lancet* 2016;388(10044):596-605. doi: 10.1016/S0140-6736(16)00342-1
27. Huffman MD, Rao KD, Pichon-Riviere A, et al. A cross-sectional study of the microeconomic impact of cardiovascular disease hospitalization in four low- and middle-income countries. *PloS one* 2011;6(6):e20821. doi: 10.1371/journal.pone.0020821
28. Rao K, Bhatnagar A, Murphy A. Socio-economic inequalities in the financing of cardiovascular & diabetes inpatient treatment in India. *Indian J Med Res* 2011;133:57-63.
29. Prinja S, Jagnoor J, Chauhan A, et al. Economic Burden of Hospitalization Due to Injuries in North India: A Cohort Study. *Int J Environ Res Public Health* 2016;13:673.
30. Karan A. Key indicators of morbidity, utilization and health expenditure - Tamil Nadu. New Dlehi: Public Health Foundation of India, 2017.
31. Kotwani A, Ewen M, Dey D, et al. Prices & availability of common medicines at six sites in India using a standard methodology. *Indian J Med Res* 2007;125(5):645-54.
32. GOI. Drug Price Control Order 1995. In: Pharmaceuticals. Do, ed. India, 1995.
33. NPPA. Drug Price Control Order 2013. India, 2016.
34. Sahay A, Jaikumar S. Does Pharmaceutical Price Regulation Result in Greater Access to Essential Medicines? Study of the impact of drug price control order on sales volume of drugs in India. Ahmedabad.: Indian Institute of Management., 2016.
35. Devadasan N, Seshadri, T., Trivedi, M., Criel, B. Promoting universal financial protection: evidence from the rashtriya Swasthya Bima Yojana (RSBY) in Gujarat, India. . *Health Res Policy Syst* 2013. ;11:1–29.
36. Prinja S, Chauhan AS, Karan A, et al. Impact of Publicly Financed Health Insurance Schemes on Healthcare Utilization and Financial Risk Protection in India: A Systematic Review. *PloS one* 2017;12(2):e0170996. doi: 10.1371/journal.pone.0170996
37. Singh PV, Tatambhotla A, Kalvakuntla R, et al. Understanding public drug procurement in India: a comparative qualitative study of five Indian states. *BMJ Open* 2013;3(2) doi: 10.1136/bmjopen-2012-001987

For peer review only

38. Ministry of Health and Family Welfare (MoHFW) GoI. NATIONAL HEALTH POLICY, 2017 2017 [Available from: https://www.nhp.gov.in//NHPfiles/national_health_policy_2017.pdf.

Figure legend:

Figure 1: Frequency and monthly per person (a) total OOP and (b) medicine OOP spending on select disease conditions, 2014

Figure 2: Monthly per person total OOP payment, medicine OOP payment and consumption expenditure

For peer review only

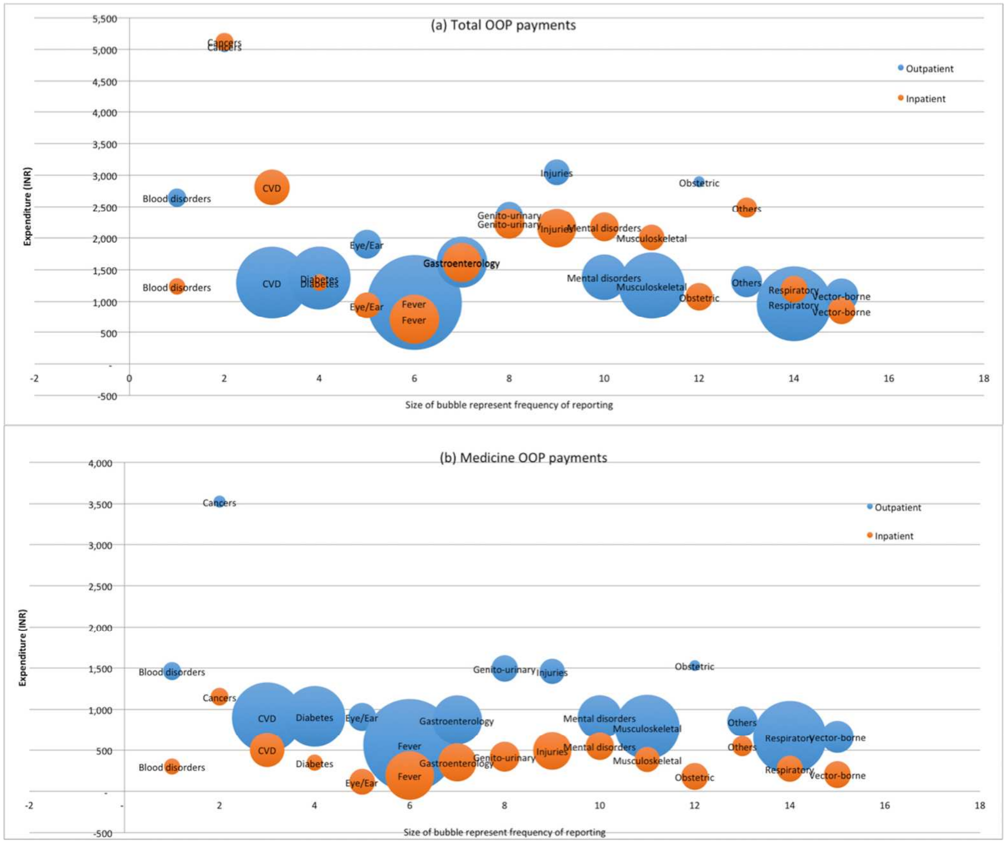

Frequency and monthly per person (a) total OOP and (b) medicine OOP spending on select disease conditions, 2014

228x190mm (300 x 300 DPI)

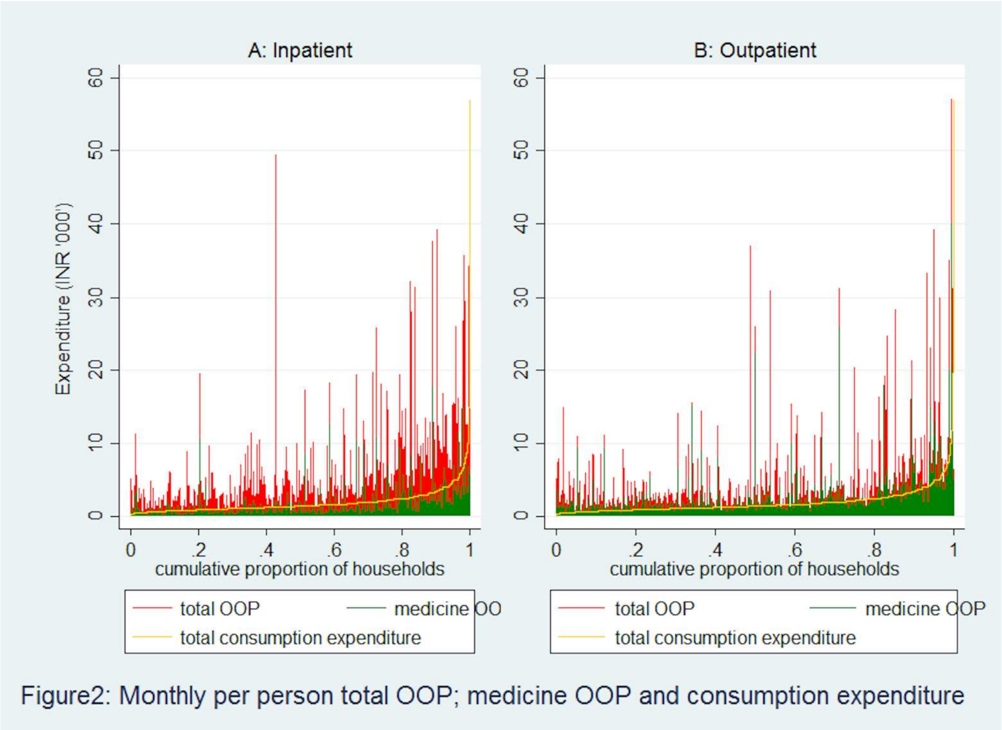

Monthly per person total OOP payment, medicine OOP payment and consumption expenditure

302x219mm (300 x 300 DPI)

Annexure

Estimation of out-of-pocket expenditure indicators and related indicators

a. Out-of-pocket payments

Total out-of-pocket (OOP) payment has been defined as the summation of all kinds of direct expenditure on purchase of medical care including expenditure on family planning devices and transportation costs to access medical care by households either as inpatient or outpatient. According to the NSSO household questionnaire the main item of expenditure considered for inpatient and outpatient are presented in Table A-I.

Table A-I: Main items of expenditure considered for inpatient and outpatient in the NSSO questionnaire

**Inpatient**		**Outpatient**	
**Heads of expenditure**	**Item code in NSSO questionnaire**	**Heads of expenditure**	**Item code in NSSO questionnaire**
medicine	410	medicine	420
X-ray, ECG, pathological test, etc.	411	X-ray, ECG, pathological test, etc.	421
doctor's/surgeon's fee	412	doctor's/ surgeon's fee	422
hospital & nursing home charges	413	family planning devices	423
other medical expenses	414	other medical expenses	424
**Total inpatient**	**419=410-414**	**Total outpatient**	**429=420-424**

The reference period of inpatient and outpatient expenditure in the consumer expenditure surveys are 1 year and 1 month respectively. Based on the information presented in Table A-I, total inpatient expenditure, outpatient expenditure and total OOP expenditure were estimated by converting inpatient expenditure for one month. Accordingly, households with any OOP have been defined as households reporting positive OOP (OOP>0) either as inpatient or outpatient or both.

b. Per person monthly OOP and OOP share

For peer review only - http://bmjopen.bmj.com/site/about/guidelines.xhtml

Per person monthly OOP is defined as total monthly OOP divided by household size for each household.

The financial burden of health expenses by households has also been estimated in terms of OOP as a share of total household expenditure and alternatively as a share of total non-food expenditure of households..

$$Sh_{OOP} = T/\exp \quad \ldots\ldots\ldots\ldots\ldots\ldots\ldots \text{(A.1)}$$

Where, 'T' is total OOP payments and 'exp' is household total (non-food) expenditure by households.

c. Catastrophic payments and headcount

Further, OOP payments are defined as catastrophic when OOP payments as a portion of total household resources are in excess of a certain threshold. A household is said to have incurred catastrophic payments if T/exp>Z, where 'T' and OOP are the same as in equation (A.1) and 'Z' is a certain threshold. The latter is arbitrary and in general, estimates are presented for a range of values for z (5, 10, 15, 25 and 40 per cent).

Accordingly, the headcount ratio of catastrophic payment is calculated as follows:

$$cat_i = \frac{1}{n}\sum 1\left(\frac{T}{exp} > Z_i\right) \ldots\ldots\ldots\ldots\ldots\ldots\ldots\ldots\ldots\ldots\ldots\ldots \text{(A.2)}$$

Where, Cat_i is catastrophic headcounts of households with OOP share exceeding a threshold defined as 'i' per cent of total household total (non-food) expenditure, 1(.) is an indicator function, which takes the value 1 if T/exp>Zi is true and 0 otherwise; n is the number of households incurring expenditure on health for various thresholds; Z_1, Z_2, Z_3 …… are the respective thresholds of the OOP share.

d. Poverty headcount

The usual headcount ratio of poverty is calculated as:

$Gross\ HP = 1/n\ \Sigma 1\ (xi \leq PL)$ …………………………… (A.3)

where: 1(.) is a function, taking the value 1 if person belong to a household with consumption expenditure lower the value of poverty line value, x= Household total consumption expenditure; PL= Poverty Line and n is total population.

Headcount of poverty after deducting OOP from household consumption expenditure can be defined as:

For peer review only

Net HP = 1/n Σ 1 ((xi-T)≤ PL). ……………………………… (A.4)

where: T= per capita OOP

The OOP induced poverty headcount finally is estimated as: (*Gross HP - Net HP).*

e. Poverty gap

Poverty gap is defined as difference between values of poverty lines and household consumption expenditure for the poor as defined in the sub-section d above. Using the household level data poverty gap for the poor is estimated as follows:

Gross poverty gap = 1/n Σ 1 (xi- PL)| if i=poor …………………………… (A.5)

In equation A.5 'xi-PL' is the difference between household expenditure per person and poverty line. For all positive values of this difference mean gap is estimated. Finally, poverty gap net of OOP payments is estimated as follows:

Net poverty gap = 1/n Σ 1 ((xi-T)- PL)| if i=poor after netting OOP ……… (A.6)

Table A-II. Prevalence and average per episode total and medicine out of pocket payments by disease conditions in 2014

	Outpatient 15 days recall						Inpatient 365 days recall					
	Prevalence		Per episode		estimated monthly		Prevalence		Per episode		estimated monthly	
Ailment	Number of episodes	%	Total OOP	Drug	Total OOP	Drug	Number of episodes	%	Total OOP	Drug	Total OOP	Drug
Fever	2,71,43,431	22.71	488	280	975	561	74,12,043	12.95	8670	2329	713	191
TB/Filaria/Tetanus	11,08,425	0.93	524	287	1047	573	6,14,933	1.07	14731	4134	1,211	340
STD/HIV/AIDS	1,20,714	0.1	538	268	1076	536	88,935	0.16	6906	1633	568	134
Vector-borne	33,33,651	2.79	549	332	1097	663	22,65,189	3.96	10288	2460	846	202

Cancers	4,52,513	0.38	2527	1763	5054	3526	9,78,764	1.71	62297	14037	5,121	1,154
Blood disease	10,26,129	0.86	1322	731	2643	1463	8,10,752	1.42	15035	3650	1,236	300
Diabetes	1,17,55,081	9.84	683	456	1367	911	8,17,199	1.43	15746	4224	1,294	347
Other Metabolic	20,50,282	1.72	712	340	1423	679	2,60,707	0.46	15429	3600	1,268	296
Mental disorders	61,56,374	5.15	690	451	1380	902	24,87,836	4.35	26428	6685	2,172	549
Eye/Ear	24,67,286	2.06	950	454	1899	908	20,82,420	3.64	11350	1407	933	116
Cardiovascular diseases	1,55,65,223	13.02	645	449	1289	899	37,82,374	6.61	34167	6129	2,808	504
Respiratory diseases	1,69,58,670	14.19	478	328	955	656	21,40,762	3.74	14491	3325	1,191	273
Gastroenterlogy	77,13,330	6.45	809	434	1617	869	45,40,520	7.93	19587	4260	1,610	350
Skin	28,34,892	2.37	522	370	1043	740	3,80,346	0.66	12123	3514	996	289
Musculo-Skeletal	1,32,29,065	11.07	622	391	1244	782	19,66,211	3.44	24379	4677	2,004	384
Genito-Urinary	21,91,953	1.83	1183	747	2365	1494	28,01,133	4.89	27085	5094	2,226	419
Obstetric	3,64,060	0.3	1448	765	2896	1529	22,64,628	3.96	13050	2189	1,073	180
Injuries	19,93,646	1.67	1522	730	3045	1460	46,19,876	8.07	26242	6000	2,157	493
Others	28,92,298	2.42	655	426	1310	853	12,97,049	2.27	30196	6666	2,482	548
Childbirth	1,46,562	0.12	585	398	1169	797	1,56,17,000	27.29	8508	1729	699	142
